# MatP regulates the coordinated action of topoisomerase IV and MukBEF in chromosome segregation

**DOI:** 10.1038/ncomms10466

**Published:** 2016-01-28

**Authors:** Sophie Nolivos, Amy L. Upton, Anjana Badrinarayanan, Julius Müller, Katarzyna Zawadzka, Jakub Wiktor, Amber Gill, Lidia Arciszewska, Emilien Nicolas, David Sherratt

**Affiliations:** 1 Department of Biochemistry, University of Oxford, South Parks Road, Oxford OX1 3QU, UK; 2 The Jenner Institute, University of Oxford, Old Road Campus Research Building, Roosevelt Drive, Oxford OX3 7DQ, UK; 3 Kavli Institute of NanoScience, Lorentzweg 1, Delft 2628 CJ, The Netherlands

## Abstract

The *Escherichia coli* SMC complex, MukBEF, forms clusters of molecules that interact with the decatenase topisomerase IV and which are normally associated with the chromosome replication origin region (*ori*). Here we demonstrate an additional ATP-hydrolysis-dependent association of MukBEF with the replication termination region (*ter*). Consistent with this, MukBEF interacts with MatP, which binds *matS* sites in *ter*. MatP displaces wild-type MukBEF complexes from *ter*, thereby facilitating their association with *ori*, and limiting the availability of topoisomerase IV (TopoIV) at *ter*. Displacement of MukBEF is impaired when MukB ATP hydrolysis is compromised and when MatP is absent, leading to a stable association of *ter* and MukBEF. Impairing the TopoIV-MukBEF interaction delays sister *ter* segregation in cells lacking MatP. We propose that the interplay between MukBEF and MatP directs chromosome organization in relation to MukBEF clusters and associated topisomerase IV, thereby ensuring timely chromosome unlinking and segregation.

## Abstract (web summary)

MukBEF, the bacterial structural maintenance of chromosomes complex, is known to associate with origins of replication and topoisomerase IV. Here the authors show an association of MukBEF with MatP and replication termination regions, important for proper sister chromatid decatenation and segregation.

## Graphical abstract

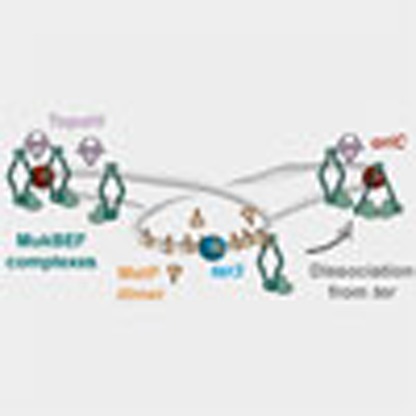

Successful segregation of newly replicated DNA requires that all interlinks between the two strands of duplex DNA be removed, as was pointed out when the structure of DNA was determined^1^. In the *Escherichia coli* chromosome, ∼450,000 links need to be removed each generation, with the two type II topoisomerases, DNA gyrase and topoisomerase IV (TopoIV) being responsible for most of this unlinking. DNA gyrase acts preferentially to remove positive supercoiling that accumulates ahead of the replication fork, while TopoIV acts potentially both in front of and behind replication forks as they proceed^2^. Inactivation of TopoIV has little or no effect on growth or DNA replication rate, but prevents segregation of newly replicated sister loci and thus cell division, thereby demonstrating its essential role in decatenation^3^.

An interaction between the ParC subunit of TopoIV and the structural maintenance of chromosomes (SMC) protein MukB has been inferred from live-cell imaging studies^4^ and demonstrated *in vitro*^5^,^6^,^7^. Furthermore, this interaction can stimulate catalysis by TopoIV *in vitro*^5^,^6^,^8^ and is predicted to promote decatenation *in vivo*^4^. MukBEF was first identified from a genetic screen designed to identify proteins that act in chromosome segregation^9^, and although it is unclear whether its primary role is in chromosome segregation or organization these functions are not mutually exclusive^10^. Functional fluorescent derivatives of MukBEF form foci that are normally associated with the replication origin region (*ori*) of the *E. coli* chromosome, and which have been proposed to position *ori*s before and after their replication, as well as recruiting TopoIV^4^,^11^,^12^. These foci contain on average eight dimers of dimer MukBEF complexes, hereafter termed MukBEF clusters, which adopt an ellipsoidal shape, with a mean diameter of ∼400 nm, irrespective of whether MukB, MukE or MukF was labelled^13^. Cluster association with *ori* requires that MukB can bind and hydrolyse ATP efficiently and that the ATPase heads can engage^13^. MukBEF complexes are restricted to some γ- and δ-proteobacteria, and have co-evolved with a subset of genes, including *matP*, whose product binds to multiple *matS* sites in the replication termination region (*ter)* and organizes the Ter macrodomain^14^,^15^,^16^.

Here we demonstrate an interaction between *E. coli* MukB and MatP by ChIP-seq, *in vitro* biochemistry and an *in vivo* two-hybrid analysis. Cells lacking MatP show a stable association of both *ori* and *ter* with positioned MukBEF clusters, consistent with MatP directing the displacement of wild-type MukBEF complexes from *ter*. This dispacement, which is impaired when MukB ATP hydrolysis is compromised, thereby facilitates MukBEF cluster association with *ori*. Cells lacking MatP show precocious *ter* segregation, which is delayed when the TopoIV-MukBEF interaction is impaired. We propose that the interplay between MukBEF and MatP directs the positioning of the chromosome with respect to MukBEF clusters and their associated TopoIV, thereby ensuring normal chromosome organization, and timely unlinking and segregation.

## Results

### ATPase-impaired MukB^EQ^EF complexes form clusters at *ter*

**Figure 1:**
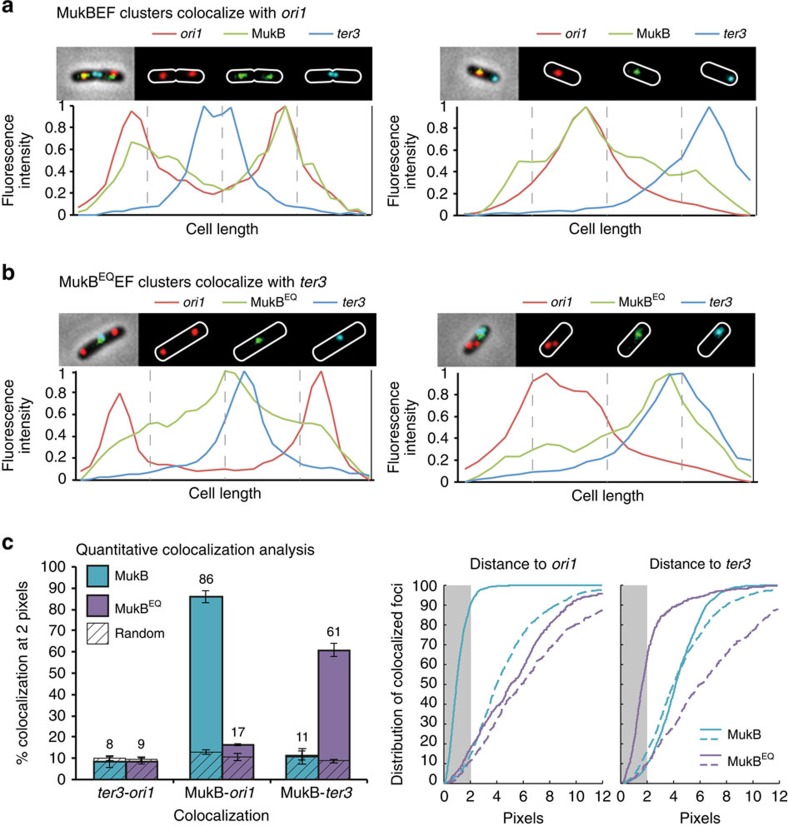
ATP hydrolysis-impaired MukB^EQ^EF complexes associate with *ter.* Localization of *ori1*, *ter3*, and MukB-mYPet (SN182) (**a**) or MukB^EQ^-mYPet (SN311) (**b**). The *ori1* locus is a *lacO* array bound by LacI-mCherry, and the *ter3* locus is a *tetO* array bound by TetR–CFP. The normalized fluorescence intensity along the cell length is shown below each image. (**c**) Left panel: % colocalization at 2 pixels between MukB, MukB^EQ^, *ori1* and *ter3* focus centroids in the combinations shown. Dashed boxes represent the distances measured to a random pixel position in the cells analysed. The histograms show the mean (±s.d.) of three independent experiments. Middle and right panels show cumulative distributions of the distances between MukB, or MukB^EQ^, and *ori1* or *ter3* foci, respectively, obtained in one experiment. Dashed lines represent distances measured to a random pixel. The grey rectangle indicates the distances within 2 pixels (258 nm). See also Supplementary Figs 1 and 2. CFP, cyan fluorescent protein).

Wild-type MukBEF complexes form clusters of molecules, revealed as fluorescent foci that associate with *ori* in steady-state cells when functional derivatives of MukB, MukE or MukF were fluorescently labelled and expressed from their endogenous promoters (Fig. 1a; refs 11, 12). To assess this association quantitatively, we measured the shortest distance between a MukBEF centroid and the centroid of the fluorescent repressor-bound *ori1* locus, located 15 kb CCW of *oriC* (Fig. 1c). This showed that in 86% of cells the distance between a MukBEF centroid and an *ori1* focus centroid was ≤258 nm (2 pixels). This distance was used to define ‘colocalization', a descriptive term that does not imply an interaction between MukBEF clusters and any specific sequence in the chromosome.

When we analysed a *mukB[E1407Q*] mutant, whose protein product (hereafter, MukB^EQ^) binds ATP, but is impaired in ATP hydrolysis^13^,^17^,^18^,^19^, we observed that the fluorescently labelled MukB^EQ^EF formed foci that colocalized with *ter3*, a locus 50 kb CW of *dif*, irrespective of whether C-terminal green fluorescent protein, mYPet or mCherry fusions were used, and whether MukB^EQ^, MukE or MukF were labelled (Fig. 1a–c; ref. 13). Unlike wild-type MukBEF, we observed little or no *ori1* colocalization with this mutant (Fig. 1c). Colocalization of MukB^EQ^EF and *ter3*, occurred when *ter3* was at midcell, where the divisome forms, or towards a cell pole, discounting the possibility that MukB^EQ^EF is divisome associated rather than directly with *ter3* (Fig. 1b). Consistent with the *ter3* association, MukB^EQ^EF focus-containing cells generally had a single focus, and quantitative analysis showed 61% colocalization between *ter3* centroids and MukB^EQ^EF centroids (Fig. 1b,c; Supplementary Fig. 1). Cells expressing MukB^EQ^EF from the endogenous locus demonstrate all of the classical Muk^−^ phenotypes—temperature-sensitive growth in rich medium, production of anucleate cells and a chromosome in which *ori* is mispositioned, as indicated by the decreased fraction of newborn cells with *ori* located at midcell and an increased fraction of cells with polar *ori*s when compared with the Muk^+^ strain (compare Fig. 1a,b; Supplementary Figs 1 and 2; refs 12, 20).

**Figure 2:**
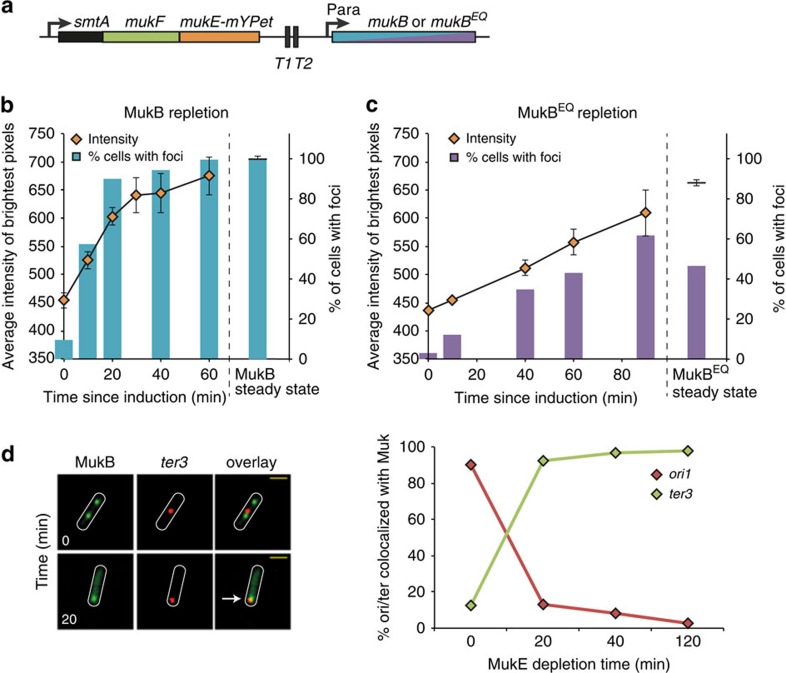
MukB^EQ^EF clusters form slowly. (**a**) Wild-type MukB or MukB^EQ^ were expressed from an arabinose-inducible promoter at the chromosomal *mukB* locus. Transcription from the *smtA* promoter was blocked by terminators T1–T2. Repletion of wild-type MukB (AU2064) (**b**) and MukB^EQ^ (AU2089) (**c**) in Δ*mukB* cells. Two methods were used to monitor focus formation; the average intensity of the brightest MukE-mYPet pixel in cells, as well as manual counting of foci (*n*=460–1,000 cells). Brightest pixel data are means (±s.e.m) of three experiments. Steady-state data were from cells where MukB/MukB^EQ^ was expressed from their normal promoters. See also Supplementary Fig. 3. (**d**) MukBEF foci colocalize with *ter* during MukE depletion. MukBEF focus position was followed before (0) and after depletion. Left: snapshot images of cells with labelled *ter3* and MukB are shown for the indicated depletion times. The arrow shows colocalization of MukB with *ter* on MukE depletion. Right: % *ori1* (Ab86) or *ter3* (Ab227) foci associated with a MukB focus, in cells with MukBEF foci, were plotted for each time point. After 80 min of depletion, cells had only weak MukBEF foci. *n*≥300.

We showed that MukB^EQ^EF has residual ATPase activity that is ∼15% of that of the wild-type complex (Supplementary Fig. 2d), in agreement with that of *Bacillus subtilis* SMC complexes^17^,^21^. Consistent with this, molecules of MukB^EQ^EF in clusters had dramatically reduced turnover in a FRAP analysis, indicating that ATP hydrolysis is required for loading onto and/or dissociation from DNA^13^. Therefore, we explored the possibility that residual ATP hydrolysis by MukB^EQ^EF might be required for its association with *ter*, but insufficient for dissociation from *ter* and association with *ori*. To investigate this, we compared repletion of wild-type MukB and MukB^EQ^ in cells that were otherwise MukB^−^E^+^F^+^. MukB or MukB^EQ^ proteins were expressed from their normal chromosomal positions, but under the control of the P_ara_ promoter rather than their endogenous promoter (Fig. 2a). The formation of MukBEF clusters was visualized after induction of expression, using MukE-mYPet (Fig. 2; Supplementary Fig. 3). The increase in intensity of the brightest MukE pixel in cells was used to measure MukBEF focus formation, along with manual counting of MukBEF/MukB^EQ^EF focus number. Expression of wild-type MukB, led to a rapid and linear increase in pixel intensity for ∼20 min, mirrored by a concomitant increase in the fraction of cells with foci (Fig. 2b). The association of MukBEF foci with *ori1* foci had approached the steady-state level after 20 min of repletion, when the relative positions of MukBEF and *ori1* foci were almost identical to those in wild-type cells (Supplementary Fig. 3). Longer repletion periods resulted in some aberrant focus positioning, indicative of excess MukB mirroring effects of MukBEF impairment, perhaps because excess MukB titrates out MukEF from functional complexes, consistent with other observations^22^.

The rate of formation of MukB^EQ^EF clusters was 3.8-fold (brightest pixel) to 6.2-fold (fraction of cells with foci) slower than for wild-type MukBEF clusters. This result indicates that the ∼15% residual ATP hydrolysis by MukB^EQ^EF is necessary for loading onto *ter*, and is consistent with the requirement of ATP hydrolysis for stable association of other SMC complexes with DNA^18^,^21^. We also showed that MukB-mYPet labelled clusters relocated from *ori* (95% colocalization at 0 min) to *ter* (98% colocalization at 20 min), as MukE was depleted by degron degradation (Fig. 2d), indicating that depletion of MukE has a regulatory consequence on MukBEF action, mirroring the MukB^EQ^EF *ter* association phenotype.

We propose that cycles of ATP binding and hydrolysis by wild-type MukBEF are required for the steady-state association of its clusters with *ori*, consistent with the FRAP analysis^13^. In contrast, a low level of ATP hydrolysis by MukB^EQ^EF complexes is sufficient for a slow but stable association with *ter*, but not sufficient for the cycles of ATP-binding and hydrolysis required for the *ori* association. The following sections reveal the molecular basis of these contrasting behaviours.

### MukBEF associates with MatP-bound *matS* sites

The contrasting cellular localization patterns of wild-type MukBEF clusters with those of MukB^EQ^EF, and those of wild type during MukE depletion, led us to analyse the association of these complexes with the *E. coli* chromosome using ChIP-seq^23^,^24^,^25^, with FLAG-tagged MukB derivatives. Although wild-type MukBEF forms clusters associated with *ori1*, we observed no significant enrichment at specific *ori* regions by ChIP-seq (Supplementary Fig. 4). Highly transcribed genes gave similar signals for MukB, MukB^EQ^, MukB^DA^ and MatP (Supplementary Fig. 4 and Supplementary Fig. 5a), most likely because single-stranded DNA present in these regions is highly reactive to formaldehyde^26^. Although enrichment at transfer RNA (tRNA) and ribosomal RNA genes has been shown for eukaryote and *B. subtilis* SMC proteins^18^,^27^,^28^, tRNA genes have been reported as false positives in yeast^29^. We have no basis for ascribing functional significance to the MukB signals in *ori* and note that unlike *B. subtilis* SMC, which interacts with ParB bound to *parS* sites, as well as to highly transcribed regions, ParB-*parS* is absent in *E. coli*, and we observed no signals that could be attributed to *parS*-like sequences.

**Figure 3:**
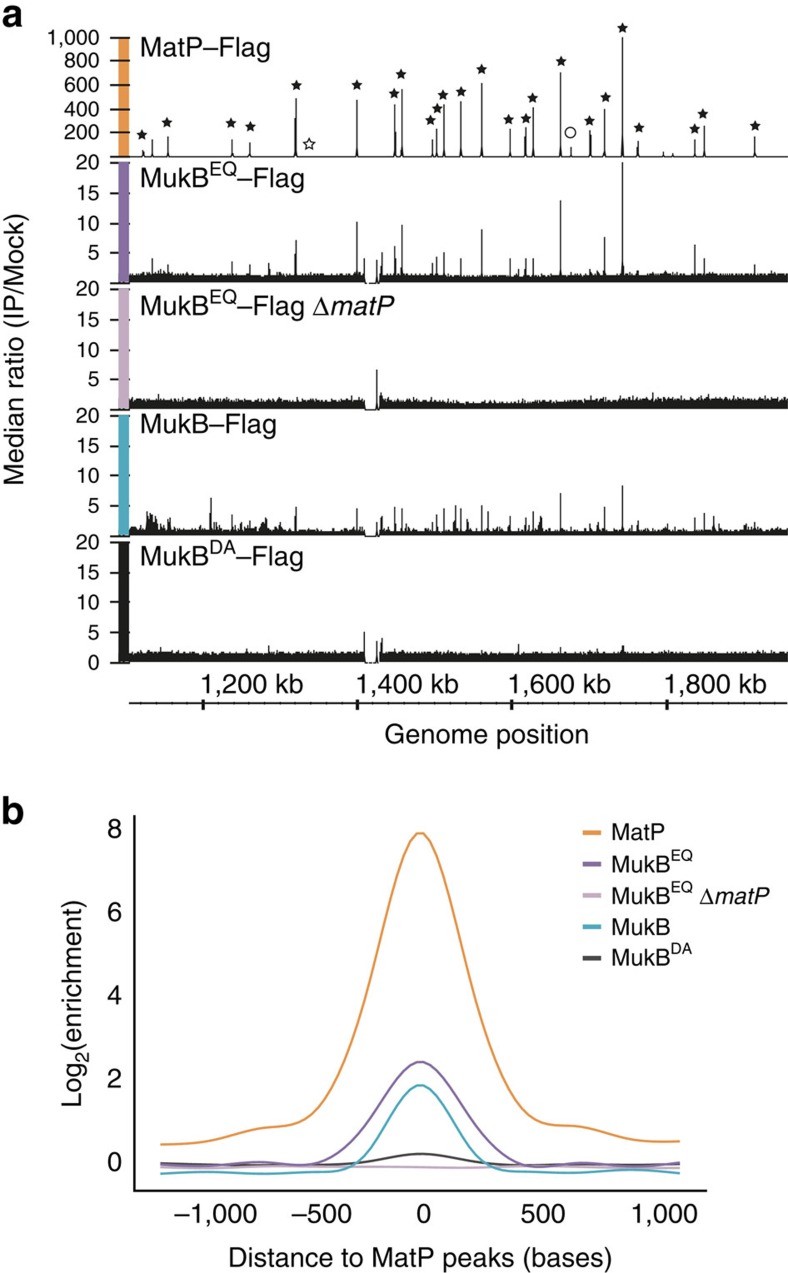
MukB^EQ^–Flag protein enrichment at *matS* sites is dependent on MatP. (**a**) Distribution of ChIP-seq signals in *ter* for MatP–Flag, MukB^EQ^–Flag, MukB^EQ^–Flag Δ*matP*, MukB–Flag and MukB^DA^–Flag. Each graph represents the ratio of the median number of reads of three experiments between Flag-tagged strains (IP) and untagged strains (Mock). Black stars indicate the positions of *matS* and pseudo *matS* sites and the circle indicates a non-*mats*-enriched region included in the analysis in **b**. *matS*5, which showed no enrichment, was not included into the analysis in **b**, and is indicated by a white star. The figure was generated using Integrated Genome Browser^56^. (**b**) Median signals of normalized samples (see experimental procedures), smoothed in a window of 2 kb centred to the position of the 26 most significant MatP peaks (indicated in **a**) are shown. The graph shows ∼1.5-fold change between MukB and MukB^EQ^. Flat signals obtained for MukB^EQ^–Flag Δ*matP* and MukB^DA^–Flag are indicative of an absence of significant enrichment. See also Supplementary Figs 4 and 5.

In contrast, when we analysed the enrichment pattern of MukB^EQ^, we observed specific signals in *ter* (Fig. 3a), consistent with the imaging analysis that shows that MukB^EQ^EF is *ter* associated (Fig. 1). The peaks obtained within *ter* were centred at *matS* sites, as revealed by motif search and by comparison of the enrichment pattern of Flag-tagged MatP and MukB^EQ^ (Fig. 3a,b; Supplementary Fig. 5b; ref. 15). In addition, the absence of MukB^EQ^ enrichment at *ter* in *ΔmatP* cells shows that the signals are MatP dependent, suggesting a physical interaction between the two proteins (Fig. 3).

By analysing the 26 chromosomal positions that gave the highest MatP enrichment, of which 21 were *matS* sites (Fig. 3a, Supplementary Fig. 5c), we observed that wild-type MukBEF exhibited a significant enrichment at these sites, while the ATP-binding deficient MukB^DA^ mutant, which showed no evidence of an association with specific regions of the chromosome by live-cell imaging^13^, was not enriched (Fig. 3b, Supplementary Fig. 5c). This indicates that MukB and MukB^EQ^ functionally interact with MatP-*matS* and are most likely loaded onto DNA in reactions that require ATP binding and hydrolysis. We propose that the relatively stable association of the ATP hydrolysis-impaired MukB^EQ^ protein with MatP-bound *matS* sites reflects the fact that efficient release of MukBEF from *ter* requires ongoing cycles of ATP hydrolysis.

Since duplex DNA is unreactive to formaldehyde *in vitro*^26^, the DNA distortion induced by MatP-binding to *matS*^30^ will enhance the formaldehyde sensitivity of *matS* sites in MatP^+^ cells. We therefore considered whether the MukBEF signals at *matS* arise because MatP-bound *matS* sites are formaldehyde reactive and that MukBEF is distributed over all of *ter*, without any specific association with MatP. We address these alternatives in the following section.

### MatP interacts with MukB *in vivo* and *in vitro*

**Figure 4:**
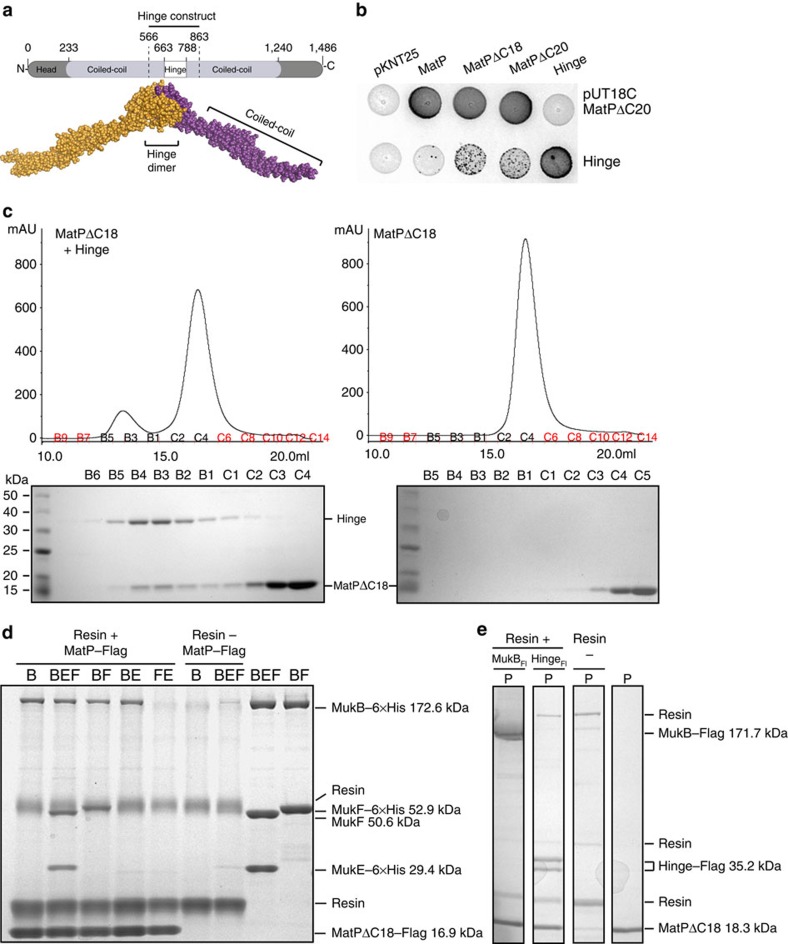
MukB and MatP interact *in vivo* and *in vitro*. (**a**) Top: schematic of MukB protein. The Hinge construct carries residues 566–863. Bottom: space-filling representation of a dimer^6^ containing most of the residues (572–854) of the Hinge construct. (**b**) The bacterial two-hybrid analysis is based on functional adenylate cyclase reconstitution^31^. Proteins of interest, as indicated, were fused to the N and the C terminus of the T25 and T18 domains, respectively. Blue colouring on X-gal plates reveals positive interactions. See also Supplementary Methods and Supplementary Fig. 6a for all combinations in MatP^+^ and Δ*matP* cells. (**c**) Size exclusion chromatography of MatPΔC18 alone (right panel), or present at seven-fold molar excess over Hinge (left panel). The elution fractions indicated in black were analysed on SDS–PAGE; shown below each graph. Because of space constraints, not all fractions are shown in the upper panels. Also see Supplementary Fig. 6b,c. (**d**) Co-Immunoprecipitation assay using resin-bound MatP–Flag, derived from over-expressed cell extracts, to assay for binding of MukB (Lane 1), MukBEF (Lane 2), MukBF (Lane 3), MukBE (Lane 4) and MukFE (Lane 5). MukB (Lane 6) and MukBEF (Lane 7) show no binding to the resin. Purified MukBEF (Lane 8) and MukBF (Lane 9), as used in the assay. The MW of the proteins used were: MukB-6 × His 172.6 kDa; MukF-6 × His 52.9 kDa; MukF 50.6 kDa; MukE-6 × His 29.4 kDa and MatPΔC18–Flag 16.9 kDa. (**e**) Co-immunoprecipitation shows MatPΔC18 is retained by MukB–Flag (lane 1) and Hinge–Flag (lane 2) attached to the anti-FLAG resin but not by the resin alone (lane 3). Lane 4: purified MatPΔC18-6 × His. Hinge proteins gave 2 bands on SDS–PAGE, maybe because of proteolytic cleavage. The MW of the proteins was: MukB–Flag 171.7 kDa; Hinge–Flag 35.2 kDa; MatPΔC18-6 × His 18.3 kDa. The lanes were derived from the same gel (shown in Supplementary Fig. 6d). MW, molecular weight.

Since a candidate for MatP dimer binding to MukBEF was the dimeric MukB hinge, we tested for a MatP-hinge interaction *in vivo* by a bacterial two-hybrid assay^31^ and *in vitro* using size exclusion chromatography and co-immunoprecipitation (Fig. 4). In the two-hybrid analysis, the MukB dimerization hinge along with 172 amino acids of associated coiled-coil (hereafter called Hinge, Fig. 4a) was tested for interactions with MatP and two truncation derivatives, of 18 and 20 C-terminal amino-acid residues (MatPΔC18 and MatPΔC20, respectively), that are unable to tetramerize^30^. We observed weak interaction signals between the Hinge and each of the MatP derivatives; these could reflect transient or weak interactions that do not require the MatP tetramerization domain (Fig. 4b). Strong signals were obtained between all combinations of MatP derivatives and between the Hinge derivatives, consistent with MatP and Hinge, each forming homodimers (Supplementary Fig. 6a).

We confirmed that the signal obtained *in vivo* reflects a direct interaction between MukB and MatP by co-immunoprecipitation and size exclusion assays. In the size exclusion assays, to maximize size differences between the proteins, we used the Hinge (36 kDa) and MatPΔC18 (18 kDa). MatPΔC18 eluted in the same fractions as the Hinge, when the Hinge was present in the sample (Fig. 4c, Supplementary Fig. 6b,c). Specific MukB–MatP interactions were also demonstrated when MatPΔC18–Flag proteins, bound to anti-Flag resin, specifically retained purified MukB, as well as MukE and MukF when they were complexed with MukB (Fig. 4d). The reciprocal experiment, in which resin-bound MukB–Flag or Hinge–Flag was used to assay for MatPΔC18 binding led to the same conclusion (Fig. 4e). We conclude that the signals of MukB enriched at *matS* sites observed in ChIP-seq reflect a physical *in vivo* interaction between the MukB hinge region and MatP.

### MatP promotes displacement of MukBEF from *ter*

**Figure 5:**
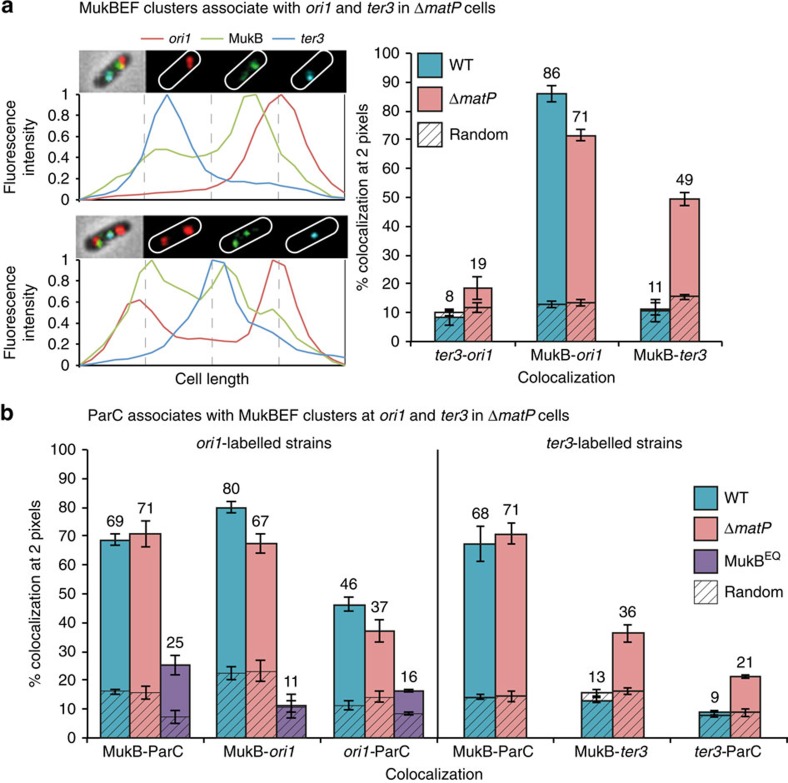
Colocalization of MukBEF complexes with TopoIV and *ter*. (**a**) Examples of colocalization of MukBEF with *ori1* and *ter3* in Δ*matP* cells (left). Δ*matP* cells have the same growth and cell cycle parameters as MatP^+^ cells (Supplementary Methods). Histograms show the colocalization between *ori1*, *ter3* and MukB-mYPet in Δ*matP* (SN302, right). *n*>2,700 cells. Wild-type data from Fig. 1 are shown for comparison. (**b**) Colocalization of MukB-mCherry, ParC-mYPet and *ori1* in wild-type (ENOX5.130), Δ*matP* (KK56) and MukB^EQ^ (ENOX5.178) cells, or *ter3* in wild-type (KK57) and Δ*matP* (KK58) strains. *n*>2,400 cells. The brightest pixel was used to localize ParC-mYPet molecules within the cell^4^. The data represent the mean (±s.d.) of three independent experiments. The percentage represents colocalization in those cells that have foci. Note that ParC-mYPet MukB^EQ^-mCherry cells grew poorly. See also Supplementary Figs 7 and 8.

Given that fluorescent MukBEF foci are normally *ori*-associated and show no *ter* colocalization (Fig. 1c), we investigated if the interaction with MatP influences the cellular localization of MukBEF clusters. In Δ*matP* cells, wild-type MukBEF foci showed a high level of colocalization with *ter3*, as well as with *ori1* (Fig. 5a). To confirm that the observed MukB-*ter* colocalization was not a consequence of increased *ter* mobility observed in the absence of MatP^15^, we repeated the analysis in *matP*ΔC*20* cells, which have lost the interactions between *matS*-bound MatP dimers, because MatPΔC20 is unable to tetramerize^30^, and which showed the increased *ter* mobility of Δ*matP* cells^30^. We infer that *ter* is no longer anchored to the divisome at midcell in these cells, because a range of assays suggests that MatPΔC20 is unlikely to interact with ZapB^30^,^32^.

The pattern of colocalization of *ori1* or *ter3* with MukBEF in *matP*ΔC*20* cells was similar to that of MatP^+^ cells (Supplementary Fig. 7). Therefore, the observed *ter3* association of MukBEF clusters in Δ*matP* cells is a direct consequence of MatP loss rather than an indirect effect arising from failure of tethering *ter* to the divisome, or from loss of MatP tetramerization-dependent condensation of *ter*. This association with *ter* in Δ*matP* cells suggests that the MukB–MatP interaction limits MukBEF cluster association with *ter*, either by limiting the accessibility to *ter*, or by promoting dissociation from *ter*. We favour the latter since MukB^EQ^EF associates with *ter* in MatP^+^ cells, making it unlikely that accessibility to *ter* is an issue (Fig. 1c).

MukB^EQ^EF clusters also remained associated with *ter3* in Δ*matP* cells, albeit with a decrease in colocalization (Supplementary Fig. 7). The difference in behaviour of MukBEF and MukB^EQ^EF with respect to their *ter3* association in MatP^+^/Δ*matP* cells suggested that wild-type MatP bound to *matS* sites promotes release of MukBEF from *ter*. This displacement of MukBEF clusters by MatP must occur inefficiently or not at all with MukB^EQ^EF clusters at *ter*, because of reduced ATP hydrolysis by these complexes, and/or because of architectural differences between the MukBEF and MukB^EQ^EF complexes. We do not know the molecular mechanism that leads to the association between *ter* and MukBEF complexes in the absence of MatP. Nevertheless, the data clearly show that this MukBEF–*ter* association leads to an interaction of MukBEF with MatP bound to *matS* sites when MatP is present. We note also that MukBEF and MukB^EQ^EF clusters associated with *ter* in the absence of MatP, like MukBEF clusters at *ori*, gave no significant ChIP-seq signal, despite forming fluorescent foci in live-cell imaging.

### MukBEF–TopoIV interact independently of chromosome position

As ParC associates with MukBEF clusters when they are positioned at *ori* (Fig. 5b; Supplementary Fig. 8; ref. 4), it was important to test whether this association was maintained when MukBEF clusters were at *ter*. The overall association between ParC and MukBEF clusters was the same in Δ*matP* and MatP^+^ cells. Furthermore, ParC colocalized with both *ter3* and *ori1* in Δ*matP* cells, maintaining its association with MukBEF clusters at both positions (Fig. 5b). Therefore, the MukBEF–TopoIV interaction occurs independently of chromosomal position.

In contrast, when we examined ParC colocalization with MukB^EQ^EF clusters at *ter*, we observed little ParC–MukB^EQ^EF association (Fig. 5b). This could be because DNA-bound MukB^EQ^EF molecules have a nucleotide state and/or architecture that fail to support stable ParC binding. Alternatively, stable association of MukB^EQ^EF with *matS*-site bound MatP could prevent ParC binding because MatP and ParC compete for binding to the MukB hinge. Irrespective of the mechanism, the nucleotide state of MukB^EQ^EF clusters loaded at *ter* influences directly, or indirectly, their association with TopoIV.

### MatP regulates MukBEF-stimulated TopoIV decatenation at *ter*

**Figure 6:**
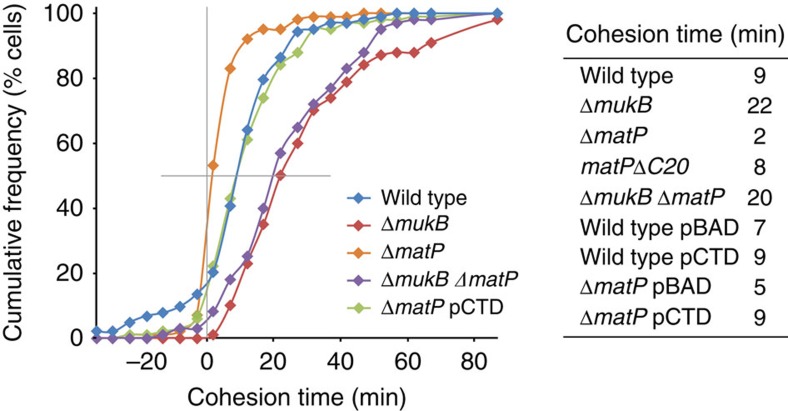
MukBEF and MatP influence *ter* segregation. Time-lapse analysis of the time between replisome (DnaN-mCherry) disappearance and *ter3* segregation. *ter3* replicates ∼2 min before termination at *terC*, and DnaN takes ∼5 min before it is unloaded after replication termination^33^; hence 7 min was added to the measured values to estimate ‘cohesion time', which we define as the time for decatenation of *ter3* after its replication. Wild type, ENOX5.212; Δ*mukB*, AU2047; Δ*matP*, AU2120; Δ*matP* Δ*mukB*, AU2125; Δ*matP* pCTD, AU2157. n≥20. See also Methods and Supplementary Fig. 9.

Since our data showed that MukBEF accesses *ter* transiently in wild-type cells, and is more stably *ter*-associated in Δ*matP* cells, we tested if the presence of functional MatP and MukBEF influences TopoIV-mediated *ter* decatenation. In time-lapse experiments, we analysed the times between disappearance of DnaN foci at replication termination and *ter3* segregation, and determined the time at which 50% of *ter3* foci had segregated. Since *ter3* replicates ∼2 min before most terminations at *terC*^3^ and DnaN molecules take ∼5 min to unload at termination^33^, the measured time underestimates the time between *ter3* replication and segregation by ∼7 min (Fig. 6 and Supplementary Fig. 9a). We therefore added 7 min to each of the measured values and defined this as ‘cohesion time', which we propose reflects the time for newly replicated *ter3* DNA to be decatenated. Consistent with this proposal, TopoIV availability at *ori1* determines the time of *ori1* segregation, with TopoIV overexpression reducing the segregation time of newly replicated *ori1* loci from 14 to 5 min^3^.

Sister *ter3* loci exhibited a 9-min cohesion time in wild-type cells, while Δ*matP* cells segregated precociously 2 min after *ter3* replication (Fig. 6 and Supplementary Fig. 9c), consistent with an increase in the fraction of cells with 2 *ter* foci in Δ*matP* cells (Supplementary Fig. 7a), as reported previously^15^. An increased fraction of 2 *ter* cells could arise as either a consequence of an increased D period in the cell cycle, or because of a precocious early separation of sister loci after replication. Flow cytometry showed no evidence of an increased D period in Δ*matP* cells (Supplementary Fig. 9d; ref. 15), supporting the hypothesis of precocious segregation in Δ*matP* cells. Furthermore, because *matPΔC20* cells have a wild-type *ter* segregation phenotype (Fig. 6 and Supplementary Fig. 9c), it seems unlikely that higher order tetramerization-dependent MatP-*matS* ‘condensed' structures delay sister separation. Therefore we propose that the precocious separation of newly replicated *ter* loci observed in Δ*matP* cells is a direct consequence of the MukBEF–TopoIV interaction at *ter* stimulating decatenation, a related observation to the demonstration that increasing TopoIV enhances decatenation at *ori1* (ref. 3). We then observed that Δ*mukB* cells had extended *ter3* cohesion times of 20–22 min, irrespective of whether cells were MatP^+^ or Δ*matP*, showing that the precocious separation in Δ*matP* cells is MukBEF dependent.

As the ParC C-terminal domain (ParC-CTD) interacts with the MukB dimerization hinge^5^,^6^,^7^, we anticipated that ParC-CTD overexpression (Supplementary Fig. 9b) would impair the ParC–MukBEF interaction and thereby allow us to test if the requirement of functional MukBEF for timely *ter* segregation is a consequence of its role in recruiting and targeting TopoIV to *ter*. Overexpression of ParC-CTD in Δ*matP* cells led to a 4-min increase in cohesion time (Fig. 6), consistent with MukBEF targeting TopoIV to preferred sites of action. In contrast, ParC-CTD overexpression did not reduce cohesion time in MatP^*+*^ cells (Fig. 6 and Supplementary Fig. 9c). The data support the hypothesis that MatP displaces wild-type MukBEF clusters and their bound TopoIV molecules from *ter*, and that the MukBEF–TopoIV association with *ter* in Δ*matP* cells promotes decatenation. A corollary is that MatP, by displacing MukBEF and its associated TopoIV, prevents premature *ter* decatenation and segregation in MatP^+^ cells. The difference in cohesion times obtained with ParC-CTD overexpression and deletion of the *mukB* gene could be a consequence of MukBEF having additional roles in *ter* segregation independently of TopoIV; for example, through chromosome positioning and/or organization. Alternatively, ParC-CTD may not compete effectively in preventing the MukBEF–ParC interaction, perhaps because this protein, unlike full-length ParC, is monomeric^34^.

## Discussion

By using an interdisciplinary approach, we have revealed how MatP regulates the position and action of *E. coli* TopoIV and MukBEF, complexes that play sequential roles in chromosome unlinking and segregation. The work has demonstrated that MatP regulates the distribution of MukBEF and TopoIV on the nucleoid by displacing MukBEF clusters and their associated TopoIV from *ter*. During replication of the *E. coli* chromosome, precatenation links between sisters arise as replication proceeds when replisomes rotate^3^,^35^. Most of these are removed by the type II topoisomerase TopoIV, although the type I topoisomerase III can potentially decatenate regions of the chromosome that are single stranded; for example, immediately behind replication forks^36^. Furthermore, FtsK DNA translocase-dependent XerCD site-specific recombination at *dif* sites also leads to decatenation^37^. Catenation links are likely to form universally as replication proceeds, since catenane accumulation has been reported when type II topoisomerase activity is compromised in a number of systems^38^,^39^.

MukBEF and other bacterial SMC complexes form clusters associated with *ori*^11^,^12^,^28^,^40^ and have been proposed to promote segregation by positioning the newly replicated sister *ori*s^4^, or by *ori* condensation^41^. The demonstration that MukB interacts with the ParC subunit of TopoIV to promote catalysis *in vitro*^5^,^6^,^7^, and that fluorescent ParC forms foci associated with MukBEF clusters at *ori in vivo*^4^, suggested a functional link between the action of TopoIV in decatenation and the facilitation of chromosome segregation by MukBEF. We have now validated such a link by showing that MukBEF-directed association of TopoIV at *ter* influences its segregation efficiency, consistent with TopoIV availability influencing sister segregation timing^3^. We propose that this is a consequence of enhanced decatenation by TopoIV, because the MukBEF–TopoIV interaction stimulates catalysis^4^ and/or leads to a higher local concentration of TopoIV in regions of the chromosome associated with MukBEF clusters. Similarly, a functional interaction between condensin and TopoII, the eukaryote orthologue of TopoIV, has been reported to aid decatenation in yeast^42^, while more generally, TopoII–condensin interactions have been proposed to be important for mitotic chromosome organization^38^,^43^, suggestive of evolutionary conserved associations and functions.

The formation of *ori*-associated MukBEF clusters requires that MukBEF is able to bind and hydrolyse ATP^11^,^13^. The clusters contain on average eight heads-engaged MukB dimers of dimers, with a relative stoichiometry of MukB/E/F of 4:4:2 (ref. 13). MukB^EQ^EF also forms heads-engaged clusters *in vivo* with identical stoichiometry. In contrast, complexes with unengaged heads have the 2:4:2 stoichiometry predicted from biochemical studies^19^. Mutants that are unable to engage heads, or to bind ATP, do not form clusters as judged by the lack of fluorescent foci^13^; similarly, the ATP-binding defective MukB^DA^, failed to give a MatP-dependent ChIP-seq signal at *matS* sites, reinforcing the view that loading of MukBEF complexes onto DNA requires ATP binding and hydrolysis. The slow rate of MukB^EQ^EF cluster formation at *ter* that we measured correlates with its residual ATPase activity and is consistent with a mechanism in which a low level of ATP hydrolysis is required for cluster formation at *ter*, but is insufficient for significant *ori* localization. The observation that MukBEF clusters associate with *ter* as MukE is depleted, mimicking the *mukB*^*EQ*^*EF* phenotype, is consistent with a regulatory role of MukE in MukBEF function^44^. We propose that MukB^EQ^EF clusters at *ter* have one or two molecules of ADP bound per MukB dimer, depending on whether one or two ATPs were hydrolysed during loading; in either case the observed stoichiometry would only result if the heads remained engaged as a consequence of failing to release product ADP and/or phosphate. We favour the hypothesis that one ATP is hydrolysed on loading, while the second is hydrolysed during unloading. This is consistent with the data here, and from our earlier work that showed that MukB^EQ^EF complexes are not readily dissociated from *ter*^13^. Understanding of the molecular changes that occur during cycles of ATP binding and hydrolysis is central to a molecular understanding of SMC action.

**Figure 7:**
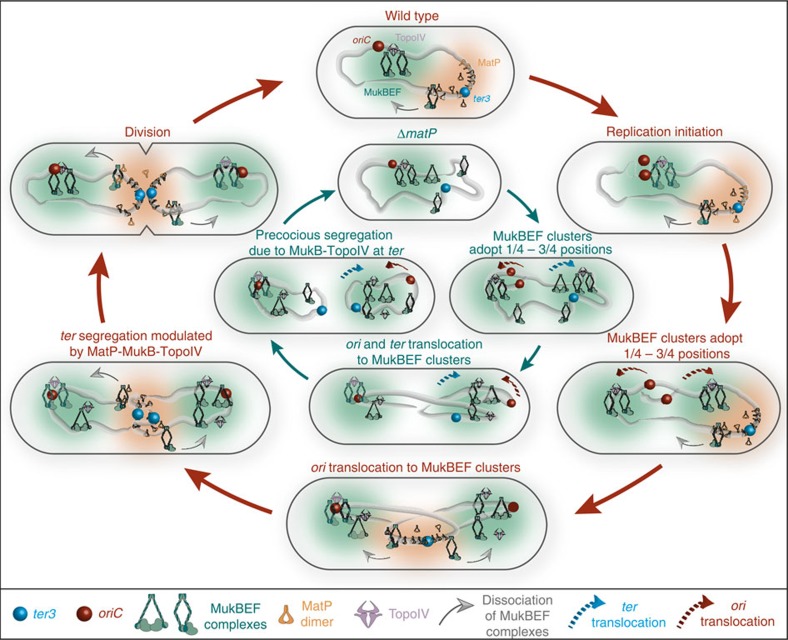
Model of the interplay between MukBEF, TopoIV and MatP in chromosome positioning, unlinking and segregation. Outer circle (red arrows; clockwise from top). In wild-type newborn cells, MukBEF clusters and associated TopoIV are positioned at midcell by an unknown mechanism. *ori* associates with these clusters. MukBEF complexes load onto DNA in ATP hydrolysis-dependent reactions. Complexes that encounter MatP-*matS* undergo ATP-hydrolysis-dependent dissociation from *ter*, thereby depleting the *ter* region from MukBEF whether in single complexes or in clusters, (and thereby facilitating the *ori* association). After replication initiation, MukBEF clusters localize to the nucleoid quarter positions, followed by the newly segregated *ori*s^4^. The process that directs the *ori* to the MukBEF clusters is unknown, but appears to require multiple cycles of ATP binding and hydrolysis, could involve repeated interactions with the chromosome by a ‘rock-walker'^13^ or ‘zipping'^47^,^48^ mechanism, and may facilitate chromosome ‘organization'. Directional ‘zipping' of the chromosome through MukBEF clusters, would result in active segregation or translocation of newly replicated *oris* to the cell quarter positions. MukBEF complexes interact with TopoIV when not associated with MatP-*matS*, thereby enhancing decatenation other than in regions bound by MatP-*matS*. Inner circle (dark arrows; clockwise from top). In Δ*matP* cells, the positioned MukBEF clusters associate with *ori* and *ter*, because the lack of MatP prevents displacement of *ter* from MukBEF complexes. Consequently, the MukBEF–TopoIV interaction leads to enhanced decatenation at *ter* and precocious segregation of newly replicated *ters*.

The functional significance of the *ter* association observed when ATPase activity was compromised in *mukB*^*EQ*^ cells, or when MukE was depleted, was reinforced when we demonstrated that in Δ*matP* cells both *ori* and *ter* colocalize with wild-type MukBEF clusters. The inferred *in vivo* interaction between MukB and MatP from the ChIP-seq analysis, and the subsequent demonstration of a direct interaction between the MukB hinge region and MatP led us to show that MatP modulates MukBEF complex activity and positioning (Fig. 7). Therefore, MatP is not a loading factor for directing the association of MukBEF clusters with *ter*, in contrast to the cohesin loading factors Scc2-4 (ref. 45), and ParB bound to *parS* sites in *B. subtilis*^28^,^40^. The mechanisms that direct the association of MukBEF clusters with *ori* and *ter* remain obscure. The MukB^EQ^EF ChIP-seq signals at *matS* correlate well with the cytological association of clusters with *ter*. We assume that the failure to see wild-type MukBEF clusters at *ter* in MatP^+^ cells by imaging is a consequence of the interaction of individual complexes being transient, yet sufficient to give a ChIP-seq signal. Our failure to identify specific binding of MukBEF to *ori* sequences is likely because the association does not distort the DNA sufficiently to make it formaldehyde sensitive and/or because MukBEF is associated with multiple unrelated DNA sequences in this region. Similarly the association of MukB^EQ^EF with *ter* in Δ*matP* cells gave no significant ChIP-seq signal in *ter*, presumably for the same reason.

We have shown that in the absence of MatP, wild-type MukBEF clusters at *ter* colocalize with ParC and this correlates directly with more efficient TopoIV-mediated *ter* decatenation in Δ*matP* cells (Fig. 7b). Modulation of TopoIV recruitment to *ter* by MukBEF and MatP may ensure sufficient cohesion time between newly replicated *ter* sisters, thereby helping coordinate the late stages of chromosome segregation with cell division. The observation that Δ*mukB* cells are more delayed in *ter* segregation than when the MukB-ParC interaction is impaired, indicates that MukBEF may have additional roles in *ter* segregation, such as positioning and/or organization, as proposed for *ori*^4^.

The results here, taken together with previous results of ourselves, and others, lead to the model depicted in Fig. 7. MukBEF complexes form positioned clusters on the nucleoid. The molecular basis for forming a cluster from MukBEF complexes is unknown, as is the positioning mechanism, but this superficially resembles the ParAB-*parS* systems that position replication origins and large protein machineries at fixed positions on the nucleoid (most commonly at mid-nucleoid and quarter positions)^46^. In steady-state cells these clusters act to exclusively position the replication origin region of the chromosome, a process dependent on the role of MatP-*matS* in displacing *ter* from MukBEF clusters.

We propose that MukBEF complexes are loaded onto different parts of the chromosome, with preferential loading within *ter*, based on the *ter* localization of clusters of MukB^EQ^EF, and of MukBEF when MatP is absent or MukE depleted. MukB^EQ^EF clusters, which associate slowly with *ter*, apparently as a consequence of their impaired ATP hydrolysis, are dissociated poorly by MatP from *ter*, indicating a requirement of ATP hydrolysis for release from *ter* and subsequent association between *ori* and these clusters. This apparent requirement of ATP hydrolysis for *ter* association and dissociation, which is consistent with FRAP data^13^, supports the hypothesis that only one of the two ATPs bound to a MukB dimer are hydrolysed in each step. The MatP-stimulated dissociation between wild-type MukBEF complexes and *ter* is apparently a requisite for subsequent exclusive association of clusters with *ori*. We propose that MukBEF complexes, whether in clusters or not, frequently transiently associate with *ter*. MatP-*matS*-promoted displacement from *ter* leads to clusters becoming excluded from *ter*, thereby facilitating *ori* association. We propose that multiple cycles of ATP binding and hydrolysis lead to translocation of MukBEF complexes in clusters with respect to the chromosome, analogous to the way that SMC complexes in *B. subtilis* have been proposed to ‘zip along the chromosome' after loading at *ori* through interaction with ParB-*parS*^47^,^48^. Such a translocation could act to actively direct *ori* to postioned MukBEF clusters. During this proposed transit, they may act as ‘condensins', since MukBEF is implicated in global chromosome organization as well as in *ori* positioning. By associating with TopoIV, the MukBEF complexes may direct decatenation activity to those regions of the nucleoid with which MukBEF interacts transiently or more stably.

The work here has highlighted the complexity of the interaction network necessary for the cell to ensure the coordination of processes responsible for chromosome organization and segregation. The organization, replication and segregation choreography of the *E. coli* chromosome in which the newly replicated origins move from the centre of the cell to the quarter positions, and *ter* migrates from the new pole to midcell to replicate^46^,^49^, implies an active positioning of *ori* and *ter*, mediated by MukBEF and MatP, respectively. The interaction between MukB and MatP, demonstrated here, brings new insight by linking *ori* and *ter*, opposite regions of the chromosome, which reside at different cellular positions for most of the cell cycle. This interplay is likely to play a central role in coordinating the early and late stages of segregation by determining the relative positions of MukBEF clusters with respect to *ori* and *ter*, thereby helping deliver TopoIV differentially to *ori* and *ter*, influencing decatenation activities spatially.

Bacterial species that have MukBEF rather than typical SMC complexes also have MatP, indicative of their co-evolved functional dependence, and reinforcing their collaboration highlighted here. As insight into the organization of bacterial chromosomes is becoming more complete, it seems there are a number of variations on common themes^50^. Future work is needed to understand the implications of having a *ter-ori*-coordinated organization in *E. coli* (with MukBEF–MatP-*matS* as key players), rather than the *ori*-coordinated (using SMC-ParAB-*parS*) organization found in bacteria in which newborn cells have *ori* at one pole and *ter* at the other.

## Methods

### Bacterial strains and plasmids and growth

Strains and plasmids used in this study are listed in Supplementary Table 1 and 2, respectively. A description of the constructions is in Supplementary Methods. Unless otherwise stated, cells were grown at 30 °C in M9 glycerol (0.2%) supplemented with required amino acids (threonine, leucine, proline, histidine and arginine—0.1 mg ml^−1^) to an *A*_600_ of 0.05–0.2 before imaging. Generation times for all strains were ∼170 min under these conditions.

### Construction of the MukB repletion strains

The MukB repletion system is similar to that used for MukF repletion^11^, however, rather than inducing MukB expression from an ectopic locus the inducible *mukB* gene was introduced at its natural chromosomal locus. The P_ara_ promoter from pBAD24 was introduced upstream of *mukB* with the T1–T2 transcriptional terminators^51^ from the *rrnB* operon upstream of this to limit transcriptional readthrough from the *smtA* promoter (Fig. 2a). When grown in glucose there should be no induction of *mukB* gene expression and the cells should have a Muk^−^ phenotype. The wild-type *araC* gene has also been re-introduced into the repletion strains to allow control of the P_ara_ promoter. The strains carry *ΔaraBAD* so that arabinose will not be metabolized.

### Live-cell imaging

Cell cultures were spotted onto an M9-gly 1% agarose pad on a slide. Images were taken using a × 100 oil immersion objective (Nikon Plan Apo × 100 1.40 PH3 DM) on a Nikon Eclipse TE2000-U microscope, equipped with a CoolSNAP HQ^2^ CCD camera (Photometrics). MetaMorph software was used for image acquisition.

### Image analysis

For the semiquantitative analysis of fluorescence distributions, fluorescence distributions in cells were plotted as line profiles using the Plot profile command of Fiji. Fluorescence intensity was normalized between 0 and 1.

For the quantitative analysis of focus localization and colocalization, detection of MukB, *ori1* and *ter3* foci was done manually using MicrobeTracker tool SpotFinderM^52^. Custom MATLAB scripts were used to extract the numbers and position of foci^53^, and to measure the shortest distance per cell between two spots (the method described in ref. 4 was modified for manually identified spots), and are available on request. In ParC-labelled strains, the analysis used the method described in ref. 4. MukB, *ori1* and *ter3* foci were detected automatically by Gaussian fitting, and the brightest pixel was used as to define a ParC focus. Data were then adjusted to represent only the fraction of cells with foci.

For the analysis of focus formation during repletion, we employed two independent methods for monitoring MukBEF focus formation using MukE-mYPet. First of all, we manually counted MukBEF foci. Second, after using MicrobeTracker to identify cells, we used a bespoke Matlab script^4^ to identify and record the intensity of the brightest mYPet pixel in every cell. The brightest pixel represents the point in the cells where fluorescent MukE-mYPet molecules have the highest residence time and thus where foci are forming. It should be noted that while MukBEF can form multiple foci within cells, the brightest pixel finds exactly one pixel with the highest intensity for MukBEF. The brightest pixel data represented in Fig. 2b,c has been adjusted to take into account that while 99% of wild-type cells have a MukBEF focus, only 47% of *mukB*^*EQ*^ cells have a MukB^EQ^EF focus in steady-state growth.

### MukB repletion in steady-state cells

Cells were grown overnight in M9 glucose at 30 °C to an *A*_600_ of 0.05. A 1 ml sample was taken, additional glucose was added to 0.2% to ensure repression of P_ara_-*mukB* and incubation continued at 30 °C for 2 h. A sample of cells (0 min) was taken and imaged before beginning the repletion. For repletion, the cells were spun down and resuspended in M9 glycerol plus 0.2% arabinose. Samples were taken at 0, 10, 20, 30, 40 and 60 min after induction of repletion and in the case of the MukB^EQ^ repletion an additional sample was taken at 90 min. All samples were spotted onto a pre-warmed M9-gly 1% agarose pad on a slide and imaged using the microscope.

### Terminus segregation in steady-state cells

Cells were grown overnight in M9 glycerol at 30 °C to an *A*_600_ of 0.05. A 1 ml sample was taken and IPTG was added to a concentration of 0.5 mM and incubated for 60 min at 30 °C. IPTG was added to induce expression of TetR-mCerulean to visualize the *ter3* array. Samples were spotted onto a pre-warmed M9-gly, 0.5 mM IPTG, 1% agarose pad on a slide and imaged in time-lapse at 30 °C. Cells were imaged every 5 min for 2 h. For analysis of asynchronously growing cells, the timing of disappearance of the replisome (mCherry-DnaN) and of *ter3* segregation was recorded. We observed that *ter3* foci can transiently separate and associate immediately after replication, presumably as a consequence of precatenation links allowing transient sister separation; hence we defined complete *ter3* separation as the point on which the foci remained separate for three consecutive 5-min time points. The robustness of the assay is indicated by the small variance in ‘cohesion times' (Fig. 6; Supplementary Fig. 9a) for a given strain as indicated by the rapid increases in 2-*ter3* focus cells. The assay interpretations assume that the different cohesion times arise from changes in segregation (decatenation) time rather than changes in time of DnaN focus disappearance.

### Bacterial two-hybrid assay

The bacterial two-hybrid assay is based on functional adenylate cyclase T18 and T25 domain reconstitution^54^. Consequent cAMP synthesis allows transcription of the lactose operon, visualized as a blue colour on X-gal indicator plates. Cells were co-transformed with pKNT25 and pUT18C plasmids expressing the proteins indicated fused to the N terminus of the T25 domain and the C terminus of the T18 domain, respectively. Co-transformation with empty plasmids was used for negative controls. Cells were incubated at 30 °C for 2 days on an LB plate containing 100 μg ml^−1^ ampicillin and 50 μg ml^−1^ kanamycin. For each transformation, 5–6 clones were resuspended in LB containing 50 μg ml^−1^ ampicillin, 50 μg ml^−1^ kanamycin and 0.5 mM IPTG and spotted on LB plates containing 50 μg ml^−1^ ampicillin, 50 μg ml^−1^ kanamycin, 0.5 mM IPTG and 60 μg ml^−1^ or 120 μg ml^−1^ X-gal. After an overnight incubation at 30 °C, plates were incubated in the dark at room temperature for 2 to 4 days to allow the colour to develop. Each transformation was performed at least three times, leading to the same overall conclusions.

### ChIP-seq

Cells were grown in LB at 37, or 22 °C for *mukB* mutant derivatives, to an *A*_600_ of 0.5, and cross-linked with 1% formaldehyde for 20 min at 22 °C. The reaction was then quenched with 500 mM glycine. The equivalent of 250 ml of cells at an *A*_600_ of 0.5 was harvested by centrifugation at 5,200*g* for 30 min, washed twice with Tris-buffered-saline (pH 7.5), resuspended in 1 ml of lysis buffer (10 mM Tris (pH 7.5), 20% sucrose, 50 mM NaCl, 10 mM EDTA, 10 mg ml^−1^ of lysozyme, 0.1 mg ml^−1^ RnaseA) and incubated at 37 °C for 30 min. Following lysis, 4 ml of immunoprecipitation buffer (50 mM HEPES-KOH (pH 7.5), 150 mM NaCl, 1 mM EDTA, 1% Triton X-100, 0.1% sodium deoxycholate, 0.1% SDS) and phenylmethylsulfonyl fluoride (final concentration of 1 mM) was added. Cellular DNA was sheared by sonication to an average size of 200–1,000 bp. Cell debris was removed by two centrifugation steps at 16,620*g* for 5 min. About 900 μl of the supernatant was used for each immunoprecipitation experiment. The sample was incubated with 2 μl of anti-Flag M2 Monoclonal Antibody (Sigma F3165) for 1 h at room temperature on a rotating wheel. Then 80 μl of DynabeadsProteinG (Life Technology) were added and incubated for 15 h at 4 °C. The beads were separated from samples by using DynaMag-2 magnet (Life Technology), and washed twice with immunoprecipitation buffer (as above), once with immunoprecipitation buffer containing 500 mM NaCl, once with wash buffer (10 mM Tris-HCl (pH 7.5), 250 mM LiCl, 1 mM EDTA, 0.5% IGEPAL CA-630, 0.5% sodium deoxycholate) and once with Tris-EDTA buffer (pH 7.5). Immunoprecipitated complexes were then removed from the beads by treatment with 100 μl of elution buffer (50 mM Tris-HCl (pH 7.5), 10 mM EDTA, 1% SDS) at 65 °C, with shaking at 1,400 r.p.m. for 30 min. Uncross-linking was achieved by addition of 90 ng μl^−1^ of proteinase K and incubation for 15 h at 50 °C followed by 4 h at 65 °C. DNA was purified using Purelink PCR Micro Kit (Invitrogen). Sequencing was performed on an Illumina HiSeq 2000 platform (Oxford Genomics Centre, Wellcome Trust Center For Human Genetics). Reads were mapped to the MG1655 reference genome and the data deposited at NCBI Omnibus^55^. See Supplementary Methods for details of the analysis.

### Biochemical assays

Recombinant proteins were subcloned into a pET vector, expressed and purified from *E. coli* C3013I. Details can be found in the Supplementary Methods. For co-immunoprecipitation assays, fresh cell lysates carrying FLAG-tagged MatPΔC18, MukB or Hinge were prepared in buffer containing 50 mM HEPES pH 7.5, 300 mM NaCl, 5% glycerol supplemented with a protease inhibitor tablet. About 10 ml of lysates were mixed with 150 μl anti-Flag M2 Affinity gel (Sigma-Aldrich), incubated for 1 h at 4 °C. The resin was then washed three times with same buffer containing 250 mM NaCl, resuspended in 1 ml of buffer I (50 mM HEPES pH 7.5, 100 mM NaCl), and purified MukB, MukE, MukF or MatPΔC18 was added as indicated. After 45-min incubation (4 °C) the resin was washed three times, resuspended in 200 μl of protein loading buffer (NEB) and analysed on 4–20% gradient SDS–PAGE. For size exclusion chromatography, purified proteins were fractionated on a Superose 6 10/300 GL column equilibrated with 50 mM HEPES, pH 7.5 buffer containing 100 mM NaCl, 1 mMDTT, 1 mM EDTA, 5 mM MgCl_2_ at a flow rate of 0.5 ml min^−1^. About 500 μl samples containing MatPΔC18 (73 μM) and/or Hinge (15 μM), were injected on the column and run at flow rate of 0.5 ml min^−1^. About 500 μl fractions were collected and analysed on 4–20% gradient SDS–PAGE.

## Additional information

**Accession codes:** ChiP-seq data are available in the NCBI's Gene Expression Omnibus^55^, series accession number GSE67221.

**How to cite this article:** Nolivos, S. *et al*. MatP regulates the coordinated action of topoisomerase IV and MukBEF in chromosome segregation. *Nat. Commun*. 7:10466 doi: 10.1038/ncomms10466 (2016).

## Supplementary Material

Supplementary Information Supplementary Figures 1-9, Supplementary Tables 1-2, Supplementary Methods and Supplementary References

## References

[b1] Watson J. D. & Crick F. H. C. Genetical implications of the structure of deoxyribonucleic acid. Nature 171, 964–967 (1953). 13063483 10.1038/171964b0

[b2] Sissi C. & Palumbo M. In front of and behind the replication fork: bacterial type IIA topoisomerases. Cell. Mol. Life Sci. 67, 2001–2024 (2010). 20165898 10.1007/s00018-010-0299-5 PMC11115839

[b3] Wang X., Reyes-Lamothe R. & Sherratt D. J. Modulation of *Escherichia coli* sister chromosome cohesion by topoisomerase IV. Genes Dev. 22, 2426–2433 (2008). 18765793 10.1101/gad.487508 PMC2532930

[b4] Nicolas E. . The SMC complex MukBEF recruits topoisomerase IV to the origin of replication region in live *Escherichia coli*. mBio 5, e01001–e01013 (2014). 24520061 10.1128/mBio.01001-13 PMC3950513

[b5] Hayama R. & Marians K. J. Physical and functional interaction between the condensin MukB and the decatenase topoisomerase IV in *Escherichia coli*. Proc. Natl Acad. Sci. USA 107, 18826–18831 (2010). 20696938 10.1073/pnas.1008140107 PMC2973858

[b6] Li Y. . *Escherichia coli* condensin MukB stimulates topoisomerase IV activity by a direct physical interaction. Proc. Natl Acad. Sci. USA 107, 18832–18837 (2010). 20921377 10.1073/pnas.1008678107 PMC2973889

[b7] Vos S. M., Stewart N. K., Oakley M. G. & Berger J. M. Structural basis for the MukB-topoisomerase IV interaction and its functional implications *in vivo*. EMBO J. 32, 2950–2962 (2013). 24097060 10.1038/emboj.2013.218 PMC3832749

[b8] Hayama R., Bahng S., Karasu M. E. & Marians K. J. The MukB-ParC interaction affects the intramolecular, not intermolecular, activities of topoisomerase IV. J. Biol. Chem. 288, 7653–7661 (2013). 23349462 10.1074/jbc.M112.418087 PMC3597806

[b9] Hiraga S. . Mutants defective in chromosome partitioning in *E. coli*. Res. Microbiol. 142, 189–194 (1991). 1925018 10.1016/0923-2508(91)90029-a

[b10] Nolivos S. & Sherratt D. The bacterial chromosome: architecture and action of bacterial SMC and SMC-like complexes. FEMS Microbiol. Rev. 38, 380–392 (2014). 24118085 10.1111/1574-6976.12045 PMC4255302

[b11] Badrinarayanan A., Lesterlin C., Reyes-Lamothe R. & Sherratt D. The *Escherichia coli* SMC complex, MukBEF, shapes nucleoid organization independently of DNA replication. J. Bacteriol. 194, 4669–4676 (2012). 22753058 10.1128/JB.00957-12 PMC3415497

[b12] Danilova O., Reyes-Lamothe R., Pinskaya M., Sherratt D. & Possoz C. MukB colocalizes with the oriC region and is required for organization of the two *Escherichia coli* chromosome arms into separate cell halves. Mol. Microbiol. 65, 1485–1492 (2007). 17824928 10.1111/j.1365-2958.2007.05881.x PMC2169520

[b13] Badrinarayanan A., Reyes-Lamothe R., Uphoff S., Leake M. C. & Sherratt D. J. *In vivo* architecture and action of bacterial structural maintenance of chromosome proteins. Science 338, 528–531 (2012). 23112333 10.1126/science.1227126 PMC3807729

[b14] Brézellec P., Hoebeke M., Hiet M.-S., Pasek S. & Ferat J.-L. DomainSieve: a protein domain-based screen that led to the identification of dam-associated genes with potential link to DNA maintenance. Bioinformatics 22, 1935–1941 (2006). 16787973 10.1093/bioinformatics/btl336

[b15] Mercier R. . The MatP/matS site-specific system organizes the terminus region of the *E. coli* chromosome into a macrodomain. Cell 135, 475–485 (2008). 18984159 10.1016/j.cell.2008.08.031

[b16] Valens M., Penaud S., Rossignol M., Cornet F. & Boccard F. Macrodomain organization of the *Escherichia coli* chromosome. EMBO J. 23, 4330–4341 (2004). 15470498 10.1038/sj.emboj.7600434 PMC524398

[b17] Hirano M. & Hirano T. Positive and negative regulation of SMC-DNA interactions by ATP and accessory proteins. EMBO J. 23, 2664–2673 (2004). 15175656 10.1038/sj.emboj.7600264 PMC449774

[b18] Hu B. . ATP hydrolysis is required for relocating cohesin from sites occupied by Its Scc2/4 loading complex. Curr. Biol. 21, 12–24 (2011). 21185190 10.1016/j.cub.2010.12.004 PMC4763544

[b19] Woo J.-S. . Structural studies of a bacterial condensin complex reveal ATP-dependent disruption of intersubunit interactions. Cell 136, 85–96 (2009). 19135891 10.1016/j.cell.2008.10.050

[b20] Niki H., Jaffé A., Imamura R., Ogura T. & Hiraga S. The new gene mukB codes for a 177 kd protein with coiled-coil domains involved in chromosome partitioning of *E. coli*. EMBO J. 10, 183–193 (1991). 1989883 10.1002/j.1460-2075.1991.tb07935.x PMC452628

[b21] Wilhelm L. . SMC condensin entraps chromosomal DNA by an ATP hydrolysis dependent loading mechanism in *Bacillus subtilis*. eLife 4, e06659 (2015). 10.7554/eLife.06659 PMC4442127 25951515

[b22] Edwards A. L., Sangurdekar D. P., Jeong K. S., Khodursky A. B. & Rybenkov V. V. Transient growth arrest in *Escherichia coli* induced by chromosome condensation. PLoS ONE 8, e84027 (2013). 24376785 10.1371/journal.pone.0084027 PMC3871593

[b23] Barski A. . High-resolution profiling of histone methylations in the human genome. Cell 129, 823–837 (2007). 17512414 10.1016/j.cell.2007.05.009

[b24] Johnson D. S., Mortazavi A., Myers R. M. & Wold B. Genome-wide mapping of in vivo protein-DNA interactions. Science 316, 1497–1502 (2007). 17540862 10.1126/science.1141319

[b25] Robertson G. . Genome-wide profiles of STAT1 DNA association using chromatin immunoprecipitation and massively parallel sequencing. Nat. Methods 4, 651–657 (2007). 17558387 10.1038/nmeth1068

[b26] McGhee J. D. & von Hippel P. H. Formaldehyde as a probe of DNA structure. r. Mechanism of the initial reaction of Formaldehyde with DNA. Biochemistry 16, 3276–3293 (1977). 19043 10.1021/bi00634a002

[b27] D'Ambrosio C. . Identification of cis-acting sites for condensin loading onto budding yeast chromosomes. Genes Dev. 22, 2215–2227 (2008). 18708580 10.1101/gad.1675708 PMC2518811

[b28] Gruber S. & Errington J. Recruitment of condensin to replication origin regions by ParB/SpoOJ promotes chromosome segregation in B. subtilis. Cell 137, 685–696 (2009). 19450516 10.1016/j.cell.2009.02.035

[b29] Teytelman L., Thurtle D. M., Rine J. & van Oudenaarden A. Highly expressed loci are vulnerable to misleading ChIP localization of multiple unrelated proteins. Proc. Natl. Acad. Sci, USA 110, 18602–18607 (2013). 24173036 10.1073/pnas.1316064110 PMC3831989

[b30] Dupaigne P. . Molecular basis for a protein-mediated dna-bridging mechanism that functions in condensation of the *E. coli* chromosome. Mol. Cell 48, 560–571 (2012). 23084832 10.1016/j.molcel.2012.09.009 PMC7505563

[b31] Karimova G., Pidoux J., Ullmann A. & Ladant D. A bacterial two-hybrid system based on a reconstituted signal transduction pathway. Proc. Natl Acad. Sci. USA 95, 5752–5756 (1998). 9576956 10.1073/pnas.95.10.5752 PMC20451

[b32] Espéli O. . A MatP-divisome interaction coordinates chromosome segregation with cell division in *E. coli*. EMBO J. 31, 3198–3211 (2012). 22580828 10.1038/emboj.2012.128 PMC3400007

[b33] Moolman M. C. . Slow unloading leads to DNA-bound β2-sliding clamp accumulation in live *Escherichia coli* cells. Nat. Commun. 5, 5820 (2014). 25520215 10.1038/ncomms6820 PMC4284645

[b34] Corbett K. D., Schoeffler A. J., Thomsen N. D. & Berger J. M. The structural basis for substrate specificity in DNA topoisomerase IV. J. Mol. Biol. 351, 545–561 (2005). 16023670 10.1016/j.jmb.2005.06.029

[b35] Joshi M. C. . Regulation of sister chromosome cohesion by the replication fork tracking protein SeqA. PLoS Genet. 9, e1003673 (2013). 23990792 10.1371/journal.pgen.1003673 PMC3749930

[b36] Perez-Cheeks B. A., Lee C., Hayama R. & Marians K. J. A role for topoisomerase III in *Escherichia coli* chromosome segregation. Mol. Microbiol. 86, 1007–1022 (2012). 23066834 10.1111/mmi.12039 PMC3549057

[b37] Grainge I. . Unlinking chromosome catenanes *in vivo* by site-specific recombination. EMBO J. 26, 4228–4238 (2007). 17805344 10.1038/sj.emboj.7601849 PMC2230843

[b38] Baxter J. ‘Breaking up is hard to do': the formation and resolution of sister chromatid intertwines. J. Mol. Biol. 427, 590–607 (2015). 25194916 10.1016/j.jmb.2014.08.022

[b39] Lucas I., Germe T., Chevrier-Miller M. & Hyrien O. Topoisomerase II can unlink replicating DNA by precatenane removal. EMBO J. 20, 6509–6519 (2001). 11707421 10.1093/emboj/20.22.6509 PMC125741

[b40] Sullivan N. L., Marquis K. A. & Rudner D. Z. Recruitment of SMC by ParB-parS organizes the origin region and promotes efficient chromosome segregation. Cell 137, 697–707 (2009). 19450517 10.1016/j.cell.2009.04.044 PMC2892783

[b41] Wang X., Llopis P. M. & Rudner D. Z. Bacillus subtilis chromosome organization oscillates between two distinct patterns. Proc. Natl Acad. Sci. USA 111, 12877–12882 (2014). 25071173 10.1073/pnas.1407461111 PMC4156703

[b42] Charbin A., Bouchoux C. & Uhlmann F. Condensin aids sister chromatid decatenation by topoisomerase II. Nucleic Acids Res. 42, 340–348 (2014). 24062159 10.1093/nar/gkt882 PMC3874195

[b43] Baxter J. & Aragón L. A model for chromosome condensation based on the interplay between condensin and topoisomerase II. Trends Genet. 28, 110–117 (2012). 22236810 10.1016/j.tig.2011.11.004

[b44] She W., Mordukhova E., Zhao H., Petrushenko Z. M. & Rybenkov V. V. Mutational analysis of MukE reveals its role in focal subcellular localization of MukBEF. Mol. Microbiol. 87, 539–552 (2013). 23171168 10.1111/mmi.12112

[b45] Ciosk R. . Cohesin's binding to chromosomes depends on a separate complex consisting of Scc2 and Scc4 proteins. Mol. Cell 5, 243–254 (2000). 10882066 10.1016/s1097-2765(00)80420-7

[b46] Reyes-Lamothe R., Nicolas E. & Sherratt D. J. Chromosome replication and segregation in bacteria. Annu. Rev. Genet. 46, 121–143 (2012). 22934648 10.1146/annurev-genet-110711-155421

[b47] Marbouty M. . Condensin- and replication-mediated bacterial chromosome folding and origin condensation revealed by hi-c and super-resolution imaging. Mol. Cell 59, 588–602 (2015). 26295962 10.1016/j.molcel.2015.07.020

[b48] Wang X. . Condensin promotes the juxtaposition of DNA flanking its loading site in Bacillus subtilis. Genes Dev. 29, 1661–1675 (2015). 26253537 10.1101/gad.265876.115 PMC4536313

[b49] Kleckner N. . The bacterial nucleoid: nature, dynamics and sister segregation. Curr. Opin. Microbiol. 22, 127–137 (2014). 25460806 10.1016/j.mib.2014.10.001 PMC4359759

[b50] Wang X. & Rudner D. Z. Spatial organization of bacterial chromosomes. Curr. Opin. Microbiol. 22, 66–72 (2014). 25460798 10.1016/j.mib.2014.09.016 PMC4359757

[b51] Orosz A., Boros I. & Venetianer P. Analysis of the complex transcription termination region of the *Escherichia coli* rrn B gene. Eur. J. Biochem. 201, 653–659 (1991). 1718749 10.1111/j.1432-1033.1991.tb16326.x

[b52] Sliusarenko O., Heinritz J., Emonet T. & Jacobs-Wagner C. High-throughput, subpixel precision analysis of bacterial morphogenesis and intracellular spatio-temporal dynamics. Mol. Microbiol. 80, 612–627 (2011). 21414037 10.1111/j.1365-2958.2011.07579.x PMC3090749

[b53] Lesterlin C., Ball G., Schermelleh L. & Sherratt D. J. RecA bundles mediate homology pairing between distant sisters during DNA break repair. Nature 506, 249–253 (2014). 24362571 10.1038/nature12868 PMC3925069

[b54] Battesti A. & Bouveret E. The bacterial two-hybrid system based on adenylate cyclase reconstitution in *Escherichia coli*. Methods 58, 325–334 (2012). 22841567 10.1016/j.ymeth.2012.07.018

[b55] Edgar R., Domrachev M. & Lash A. E. Gene Expression Omnibus: NCBI gene expression and hybridization array data repository. Nucleic Acids Res. 30, 207–210 (2002). 11752295 10.1093/nar/30.1.207 PMC99122

[b56] Nicol J. W., Helt G. A., Blanchard S. G., Raja A. & Loraine A. E. The integrated genome browser: free software for distribution and exploration of genome-scale datasets. Bioinformatics 25, 2730–2731 (2009). 19654113 10.1093/bioinformatics/btp472 PMC2759552

