# Supplementary material for: MatP regulates the coordinated action of topoisomerase IV and MukBEF in chromosome segregation
Source: Nat Commun. 2016 Jan 28;7:10466. doi: 10.1038/ncomms10466 (PMC4738335; doi:10.1038/ncomms10466)
Supplement: Supplementary Information — Supplementary Figures 1-9, Supplementary Tables 1-2, Supplementary Methods and Supplementary References [file ncomms10466-s1.pdf]

Supplementary Figures

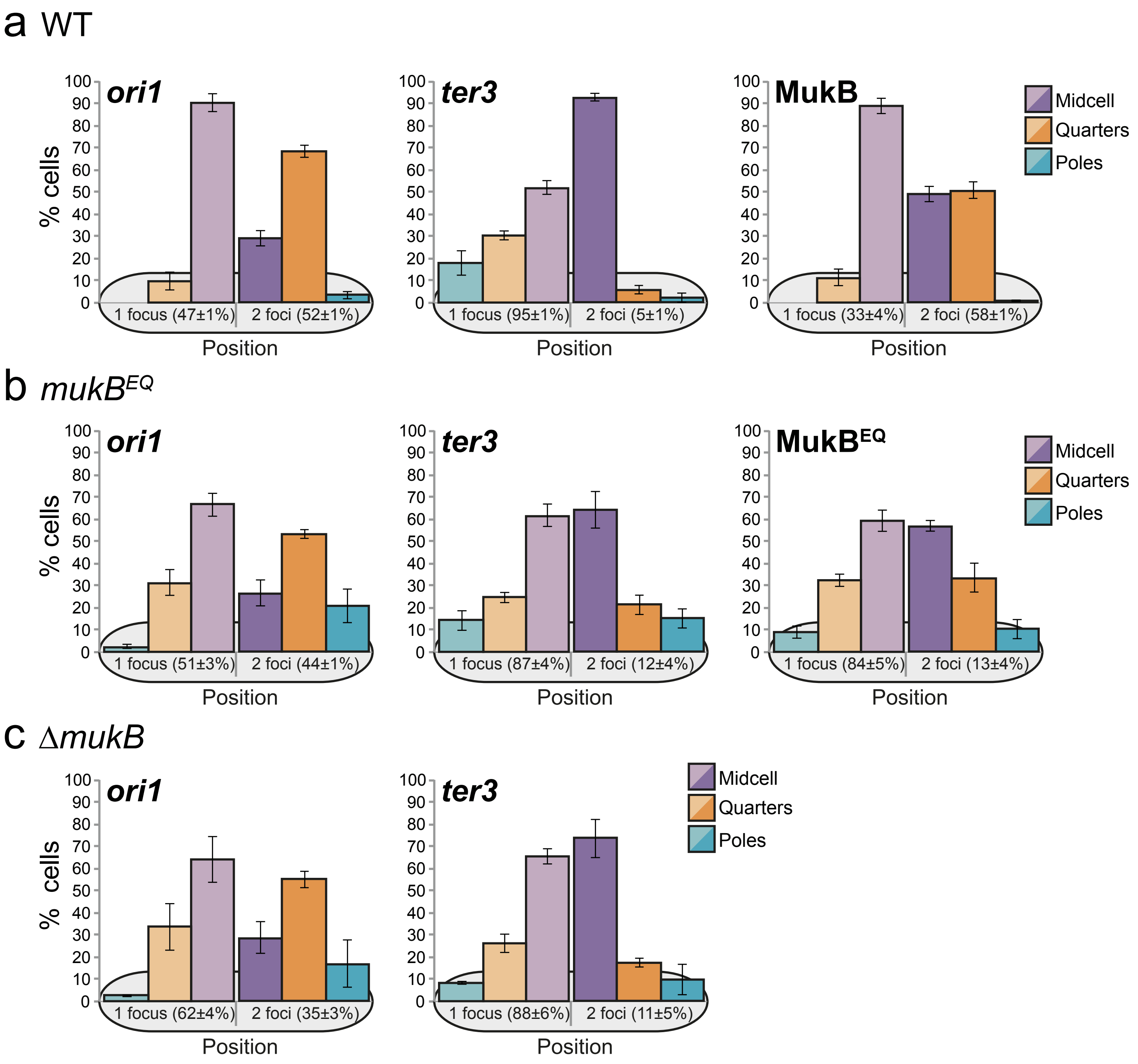

Supplementary Figure 1. Cellular position of *ori1*, *ter3* and MukB foci

Histograms represent the cellular position of foci for cells with 1 focus (left side, lightly shaded bars) and 2 foci (right side, darker bars). (**a**) Localization of *ori1*, *ter3*, and MukB-mYPet (SN182). (**b**) Localization of *ori1*, *ter3*, and MukB^{EQ}-mYPet (SN311). (**c**) Localization of *ori1* and *ter3* in Δ*mukB* strain (AU2101). The percentage of cells with 1 and 2 foci is indicated in parentheses. The data represent the mean (± s. d.) of three independent experiments (in **a** and **b**), or two independent experiments (in **c**). n > 1,500 cells.

a

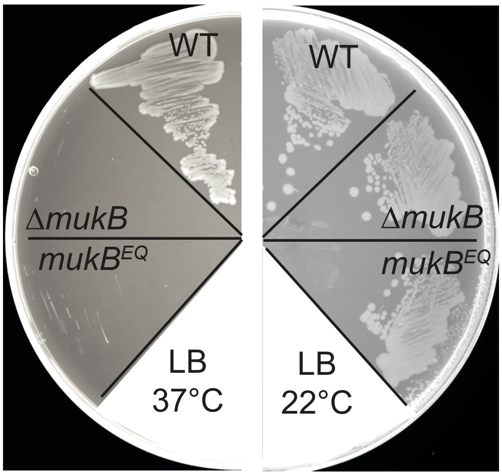

b

% anucleate cells	
WT	0.2 ± 0.3
ΔmukB	20.6 ± 5.8
mukB^{EQ}	22.2 ± 0.4

c

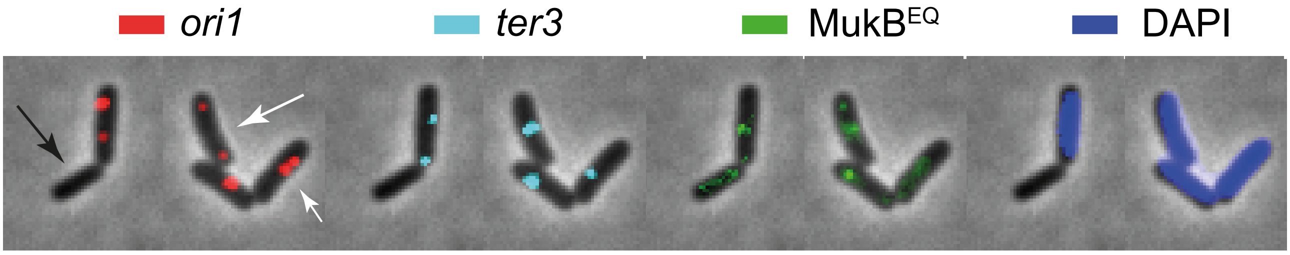

d

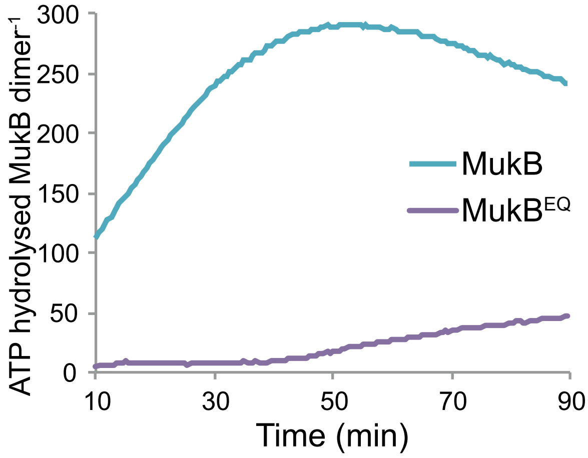

ATP hydrolysed min^{-1} dimer^{-1}	
MukB	5.63 ± 0.55
MukB^{EQ}	0.86 ± 0.11

Supplementary Figure 2. Phenotypes associated with the *mukB*^{EQ} mutation
(**a**) Comparison of growth on LB at 37°C (1 day) and 22°C (5 days). (**b**) % anucleate cells in the assay presented on Figure 1, and in a *ΔmukB* strain (AU2101). We used the absence of fluorescent *ori1* focus as measure of the % anucleate cells, after validation of the analysis on a sample using DAPI staining (**c**). The data represent the mean value (± s. d.) of three independent experiments, or two independent experiments for the *ΔmukB* strain. (**c**) Microscopy images of *mukB*^{EQ} cells (SN311) showing chromosome organization and segregation defects. Cells have been incubated 5 minutes with 1μg/ml DAPI before imaging. Black arrow indicates an anucleate cell. White arrows indicate mispositioned *ori1* foci, which can be polar (big arrow), or not well separated (small arrow). (**d**) ATPase activity of MukBEF and MukB^{EQ}EF complexes. The data have been adjusted for the residual activity measured for MukE and MukF alone. Concentrations of proteins used are MukB/MukB^{EQ} 0.5 μM; MukE 2.5 μM and MukF 1.25 μM. The number of molecules of ATP hydrolysed min^{-1} dimer^{-1} has been calculated from the linear portion of the curves. Data represented are the mean (± s. d.) of two independent experiments.

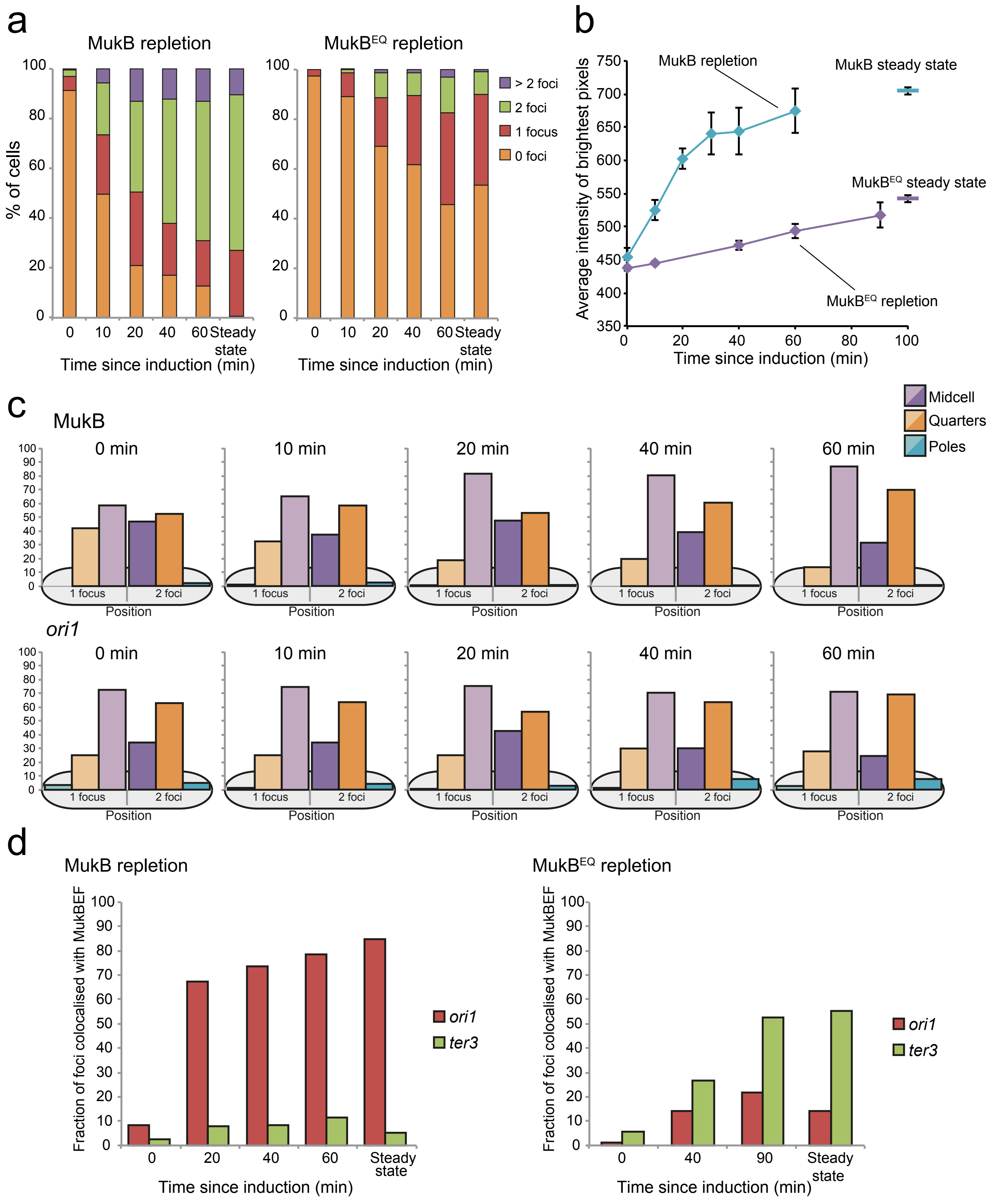

Supplementary Figure 3. MukB^{EQ}EF clusters form slowly

(**a**) Manual analysis of the number of MukBEF foci during repletion. (**b**) Raw brightest pixel data for MukB and MukB^{EQ} repletion. (**c**) Positioning of *ori1* and MukBEF foci during repletion. Foci positions were determined as being in the middle third of the cell, at the quarter positions, or at the poles. (**d**) Colocalisation of MukB and MukB^{EQ} with *ori1* and *ter3* foci during repletion.

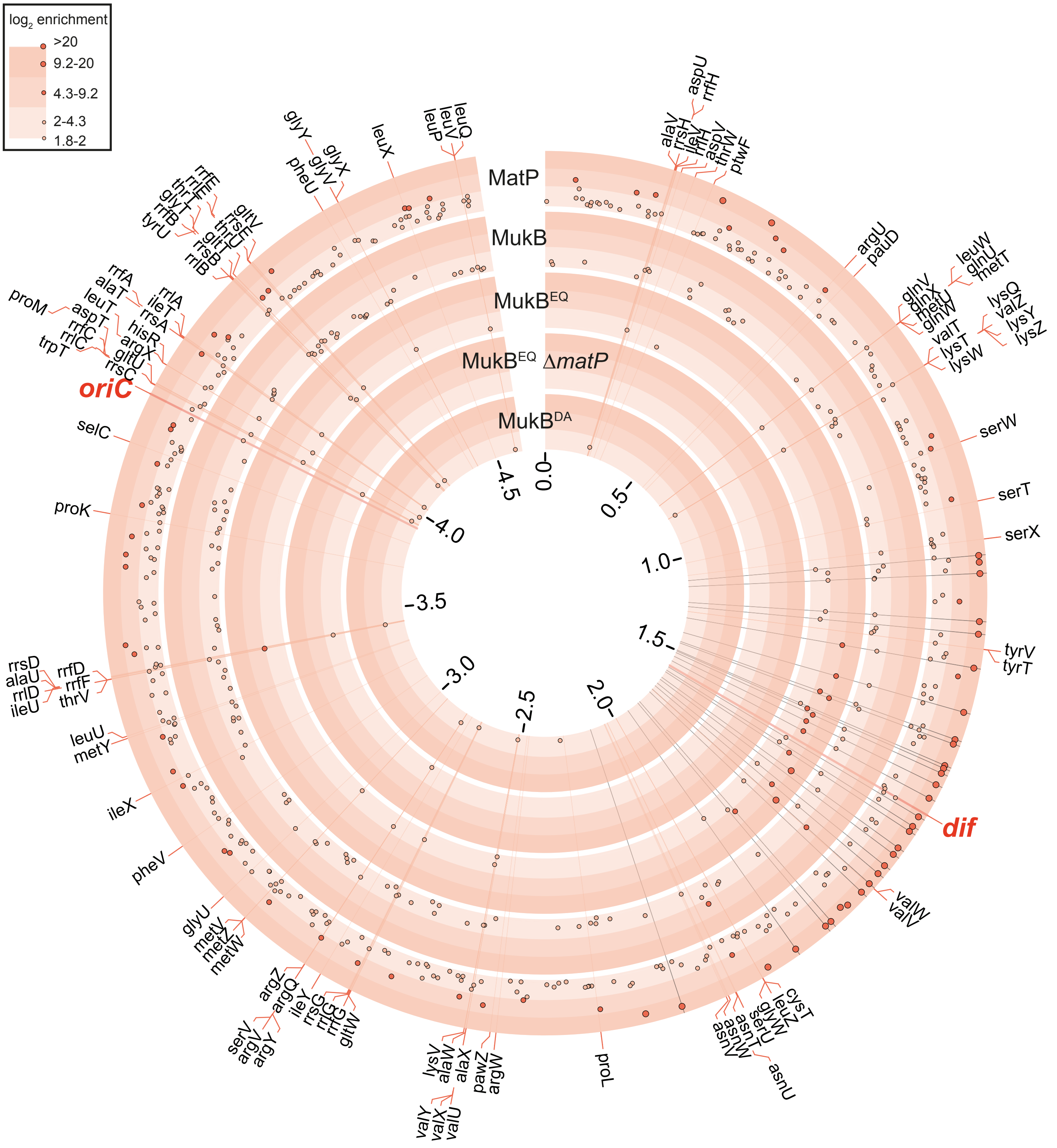

Supplementary Figure 4. ChIP-seq signal distribution along the *E. coli* chromosome
Circular representation of the enrichment (log2 IP/Mock) >1.8 for MatP-Flag, MukB-Flag, MukB^{EQ}-Flag, MukB^{EQ}-Flag Δ*matP*, and MukB^{DA}-Flag as indicated. *E. coli* chromosome coordinates are indicated in Mb. Position of the 26 MatP most enriched regions are indicated by black lines. *oriC*, *dif,* tRNA and rRNA genes, are indicated by radial red lines. The figure was generated using Circos plot[15].

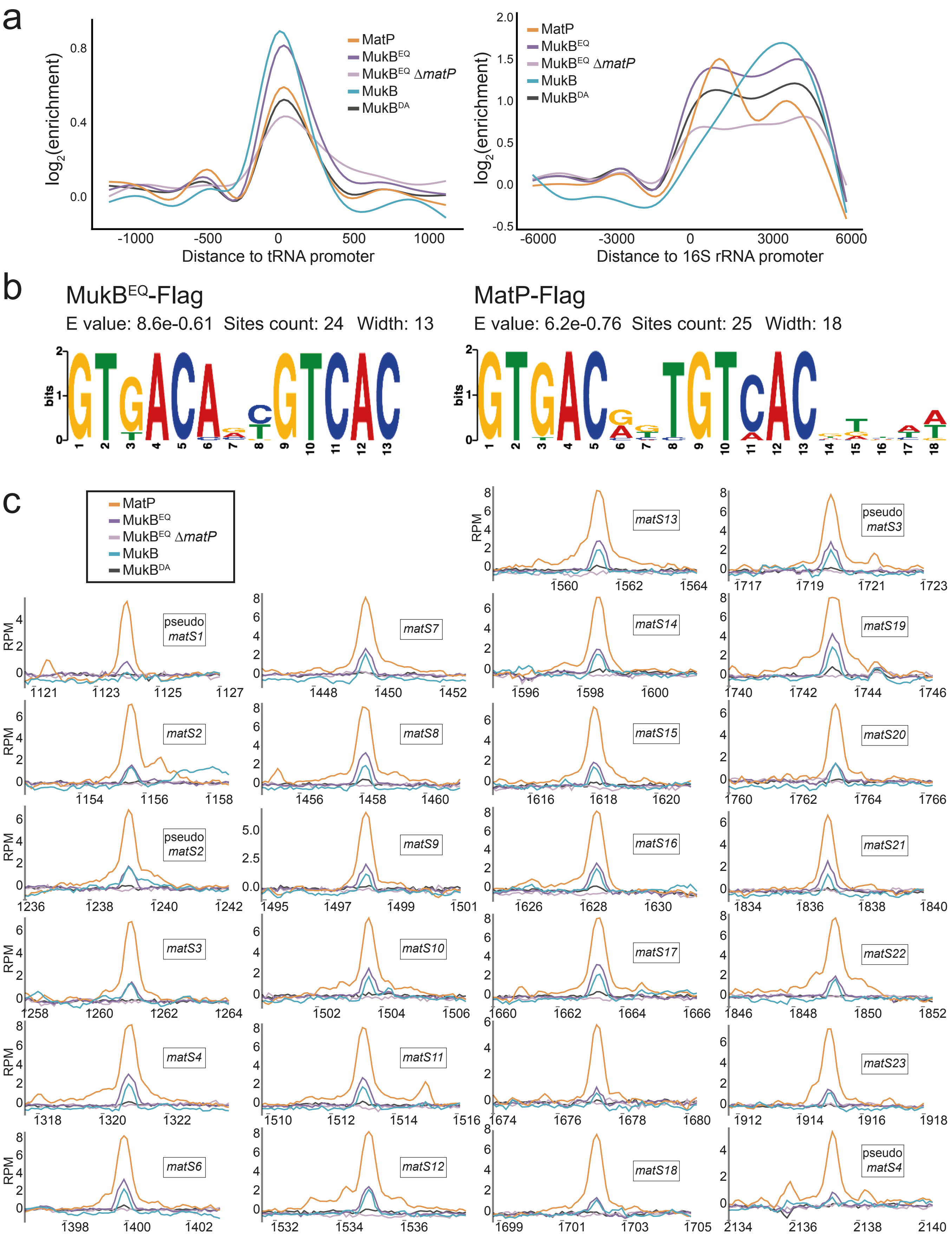

Supplementary Figure 5. MukB and MukB^{EQ}-Flag proteins are enriched at *matS* sites (**a**) Median profiles of the signal obtained at tRNA (left) and rRNA (right), like in Figure 3. (**b**) Left; most significant motif obtained for MukB^{EQ}-Flag peaks. This motif was found in 24/43 significant MukB^{EQ}-Flag peaks. Right; most significant motif obtained for MatP-Flag peaks, found in 25/26 most significant peaks. (**c**) ChIP-seq signals at the 26 MatP most enriched regions used in the Figure 3b. The numbers of reads adjusted to 1 million are plotted as a function of chromosomal position (kb).

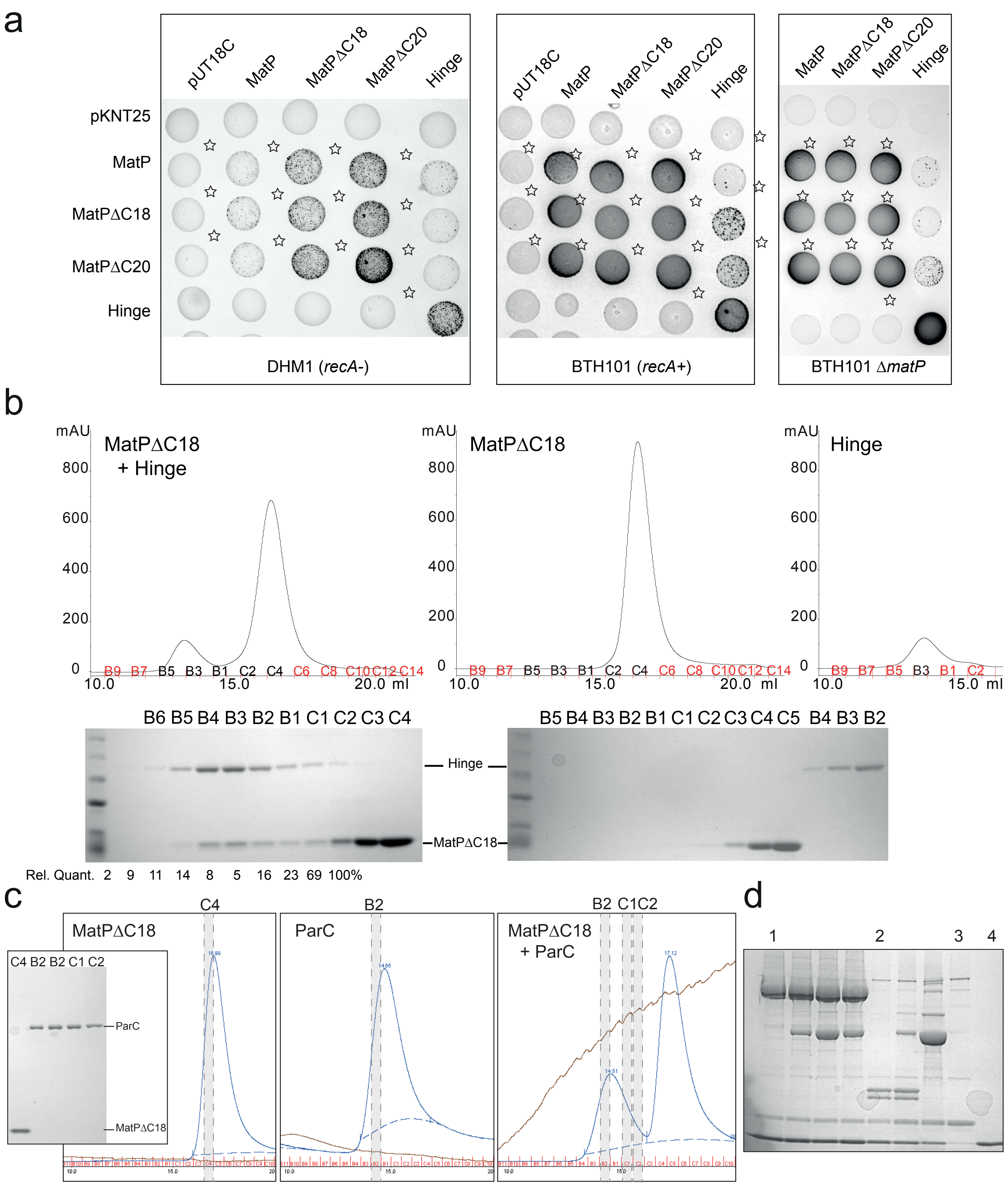

Supplementary Figure 6. MukB and MatP interact *in vivo* and *in vitro*
(**a**) Bacterial two-hybrid experiment performed as indicated with DHM1, BTH101 and BTH101Δ*matP* strains, like in Figure 4. Stars show the presence of a positive blue signal, indicative of an interaction. We note that MatP-Hinge interaction was never detected when Hinge was tagged in C-terminal with T25 domain, and MatP was tagged in N-terminal with T18 domain (**b**) Size exclusion chromatography of MatPΔC18 and Hinge analyzed together and separately, like in Figure 4. Relative quantification of MatPΔC18 when analyzed with Hinge is indicated below the SDS-PAGE gel (**c**) Size exclusion chromatography of MatPΔC18 (17μM) and ParC (5μM) analyzed together and separately, as indicated. The elutions fractions analyzed on the SDS-PAGE gel (Right panel), are indicated on top of the corresponding graph. (**d**) Uncropped gel from Figure 4e.

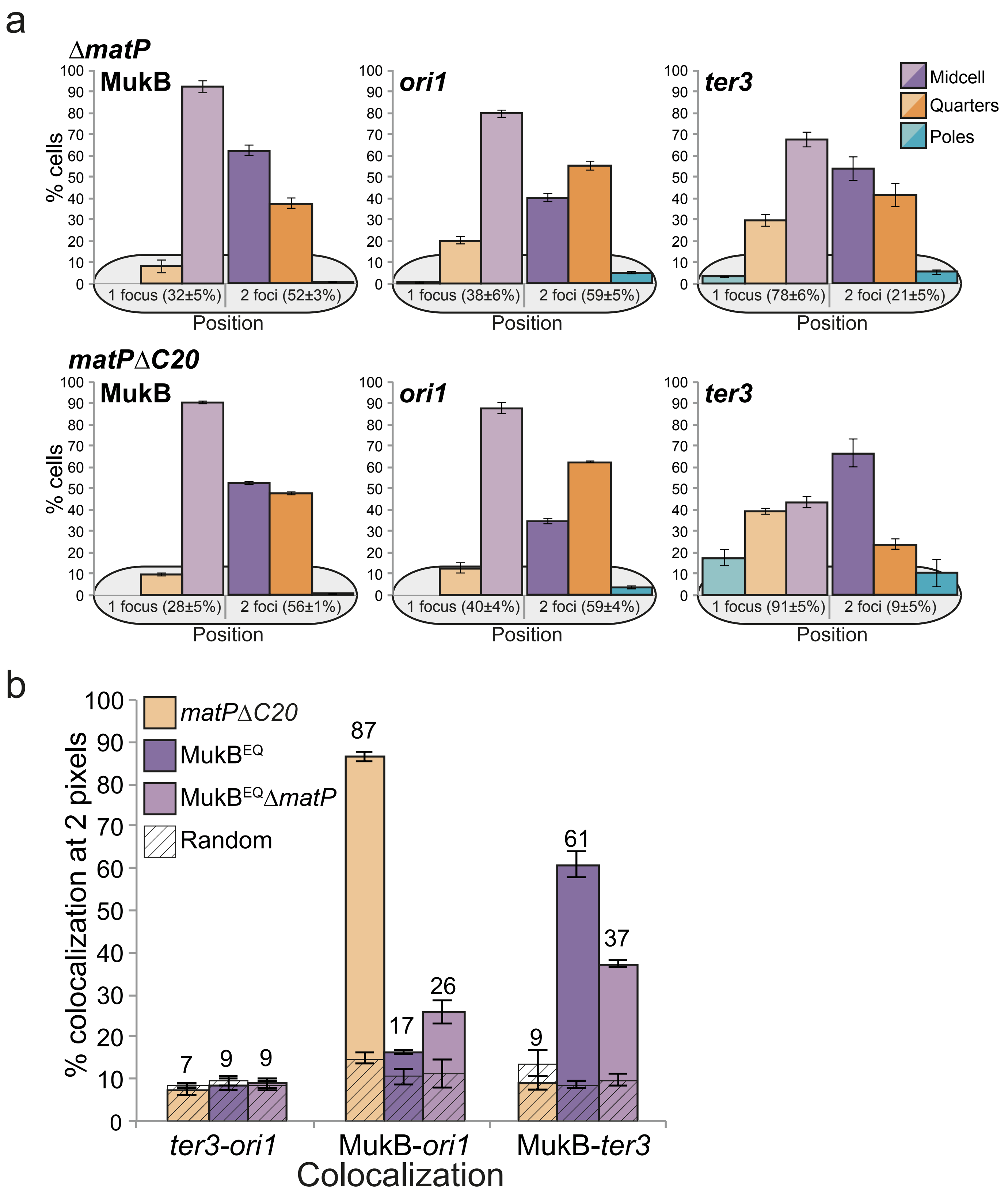

Supplementary Figure 7. Colocalization of MukBEF complexes with *ter*
(**a**) Histograms of the cellular position and distribution of MukB, *ori1* and *ter3* foci in Δ*matP* (SN302) and *matP*ΔC20 (SN399) cells (mean (± s. d.) of three independent experiments). (**b**) Colocalization between *ori1*, *ter3* and MukB-mYPet in *matP*ΔC20 (SN399) and *mukB*^{EQ} Δ*matP* cells (SN317). MukB^{EQ} data from Fig. 1 are shown for comparison. n>2,600 cells

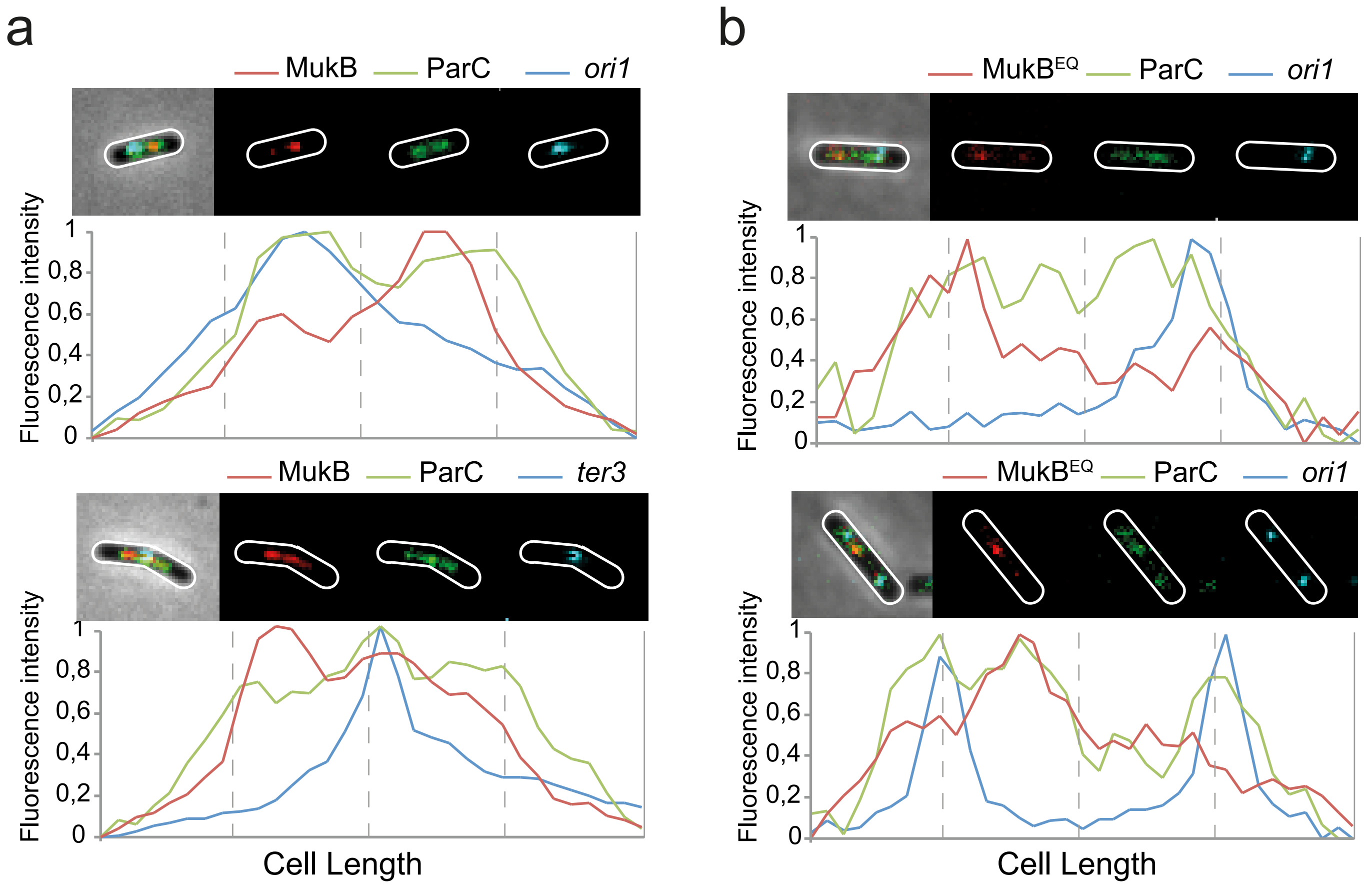

Supplementary Figure 8. ParC colocalizes with MukBEF clusters at *ori* and *ter* but poorly with MukB^{EQ}EF

(**a**) Microscopy images of MukB-mCherry, ParC-mYPet and *ori1* (top) or *ter3* (bottom) foci in Δ*matP* cells. Cell outlines are indicated in white. The normalized intensity of fluorescence along the cell length is represented below each microscopy image. (**b**) Microscopy images of MukB^{EQ}-mCherry, ParC-mYPet and *ori1*, as in **a**.

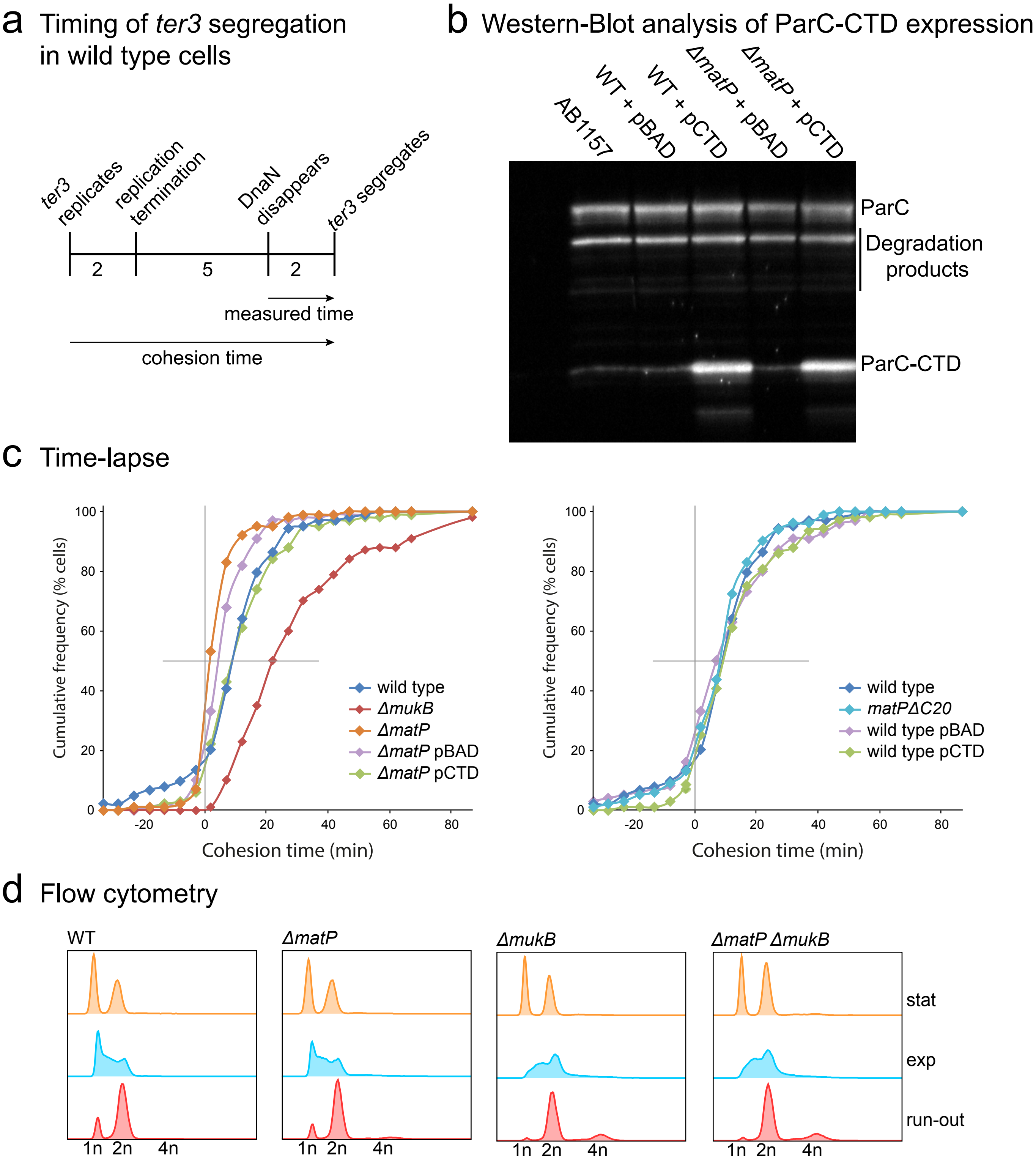

Supplementary Figure 9. MukBEF influences *ter* segregation.
(**a**) Schematic of events during wild type *ter s*egregation. *ter3* is expected to be replicated 2 min before replication termination and DnaN unloads ~5 min after termination[33]. The assays for cohesion time assume that the rate of replisome disappearance after replication termination is constant for all strains used. (**b**) Western-Blot confirming expression of the ParC-CTD construct. Polyclonal rabbit anti-ParC antibodies were used for detection of ParC and ParC-CTD. (**c**) Time-lapse analysis of the time between *ter3* replication and *ter3* segregation. Wild type (ENOX5.212); *ΔmukB* (AU2047); *ΔmatP* (AU2120); *ΔmatP* pBAD24 (AU2155); *ΔmatP* pCTD (AU2157); *matPΔC20* (AU2139); WT pBAD24 (AU2143); WT pCTD (AU2145). (**d**) Flow cytometry for wild type (ENOX5.212); *ΔmukB* (AU2047); *ΔmatP* (AU2120), and *ΔmatP ΔmukB* (AU2125) cells.

Supplementary Table 1. Bacterial strains

Strain	Relevant genotype[a]	Source or reference[a]
**AB1157 and derivatives**		
AB1157	F^-, λ^-, rac^-, *thi-1, hisG4, Δ(gpt-proA)62, argE3, thr-1, leuB6, kdgK51, rfbD1, araC14, lacY1, galK2, xylA5, mtl-1, tsx-33, supE44(glnV44), rpsL31(strR), qsr'-0, mgl-51*	1
Ab16	*mukB-mYPet-kan*	2
Ab18	*mukE-mYPet-kan*	2
Ab86	*mukE-degron frt, mukB-mYPet-Kn, ΔsspB,* P_{ara}*-sspB, tetO240::gen* at *ori1*, pWX9	3
Ab142	*lacO240::hyg* at *ori1, tetO240::gen* at *ter3,* P_{lac}*-lacI-mCherry* at *leuB,* P_{lac}*-tetR-mCerulean frt* at *galK, mukE-mYPet-kan*	RRL189 × P1.Ab18 to Km^r
Ab148	*lacO240::hyg* at *ori1, tetO240::gen* at *ter3,* P_{lac}*-lacI-mCherry frt* at *leuB,* P_{lac}*-tetR-mCerulean frt* at *galK, mukE-mYPet frt*	Derivative of Ab142, *kan* removed via pCP20
Ab198	*mukB(E1407Q)-mYPet-kan*	2
Ab227	*mukE-degron frt, mukB-mYPet-Kn, ΔsspB,* P_{ara}*-sspB, tetO240::gen* at *ter3*, pWX9	This work
AU2036	*mukE-mYPet, kan-T1-T2-*P_{ara}*-mukB*	This work
AU2047	*mCherry-DnaN,* P_{lac}*-tetR-mCerulean* at *galK frt, tetO240::gen* at *ter3, ΔmukB::kan*	ENOX5.212 × P1.AU2094 to Km^r
AU2052	*lacO240::hyg* at *ori1, tetO240::gen* at *ter3,* P_{lac}*-lacI-mCherry frt* at *leuB,* P_{lac}*-tetR-mCerulean frt* at *galK, mukE-mYPet frt, ΔaraBAD::cat (araC+)*	Ab148 × P1.RRL386 to Cm^r
AU2057	*lacO240::hyg* at *ori1, tetO240::gen* at *ter3,* P_{lac}*-lacI-mCherry frt* at *leuB,* P_{lac}*-tetR-mCerulean frt* at *galK, mukE-mYPet, ΔaraBAD::cat (araC+), kan-T1-T2-*P_{ara}*-mukB*	AU2052 × P1.AU2036 to Km^r
AU2064	*lacO240::hyg* at *ori1, tetO240::gen* at *ter3,* P_{lac}*-lacI-mCherry frt* at *leuB,* P_{lac}*-tetR-mCerulean frt* at *galK, mukE-mYPet, ΔaraBAD frt (araC+), frt T1-T2-*P_{ara}*-mukB*	Derivative of AU2057, *kan* & *cat* removed via pCP20
AU2079	*lacO240::hyg* at *ori1, tetO240::gen* at *ter3,* P_{lac}*-lacI-mCherry frt* at *leuB,* P_{lac}*-tetR-mCerulean frt* at *galK, mukE-mYPet, ΔaraBAD frt (araC+), frt T1-T2-*P_{ara}*-mukB(E1407Q)-kan*	This work
AU2084	*lacO240::hyg* at *ori1, tetO240::gen* at *ter3,* P_{lac}*-lacI-mCherry frt* at *leuB,* P_{lac}*-tetR-mCerulean frt* at *galK, mukE-mYPet, ΔaraBAD frt (araC+), frt T1-T2-*P_{ara}*-mukB(E1407Q)-kan, pRC7(mukB+)*	pRC7(*mukB*+) × AU2079 to Ap^r
AU2089	*lacO240::hyg* at *ori1, tetO240::gen* at *ter3,* P_{lac}*-lacI-mCherry frt* at *leuB,* P_{lac}*-tetR-mCerulean frt* at *galK, mukE-mYPet, ΔaraBAD frt (araC+), frt T1-T2-*P_{ara}*-mukB(E1407Q)-kan*	AU2064 × P1.AU2084 to Km^r
AU2094	*ΔmukB::kan, pRC7(mukB+)*	pRC7(*mukB*+) × RRL149 to Ap^r

AU2101	*lacO240::hyg* at *ori1, tetO240::gen* at *ter3,* P_{lac}*-lacI-mCherry frt* at *leuB,* P_{lac}*-tetR-mCerulean frt* at *galK, ΔmukB::kan*	RRL189 × P1.AU2094 to Km^r
AU2116	*lacO240::hyg* at *ori1, tetO240::gen* at *ter3,* P_{lac}*-lacI-mCherry frt* at *leuB,* P_{lac}*-tetR-mCerulean frt* at *galK, mukE-mYPet frt, mukB[E1407Q]-kan*	This work
AU2120	*mCherry-DnaN,* P_{lac}*-tetR-mCerulean frt* at *galK, tetO240::gen* at *ter3, ΔmatP::cat*	ENOX5.212 × P1.SN205 to Cm^r
AU2125	*frt mCherry-DnaN,* P_{lac}*-tetR-mCerulean frt* at *galK, tetO240::gen* at *ter3, ΔmatP::cat, ΔmukB::kan*	AU2120 × P1.AU2094 to Km^r
AU2127	*frt mCherry-DnaN,* P_{lac}*-tetR-mCerulean frt* at *galK, tetO240::gen* at *ter3, ΔaraBAD::cat (araC+)*	ENOX5.212 × P1.RRL386 to Cm^r
AU2129	*matPΔC20-cat*	This work
AU2133	*frt mCherry-DnaN,* P_{lac}*-tetR-mCerulean frt* at *galK, tetO240::gen* at *ter3, ΔaraBAD frt (araC+)*	Derivative of AU2127, *cat* removed via pCP20
AU2139	*frt mCherry-DnaN,* P_{lac}*-tetR-mCerulean frt* at *galK, tetO240::gen* at *ter3, matPΔC20-cat*	ENOX5.212 × P1.AU2129 to Cm^r
AU2143	*frt mCherry-DnaN,* P_{lac}*-tetR-mCerulean frt* at *galK, tetO240::gen* at *ter3, ΔaraBAD frt (araC+), pBAD24*	pBAD24 × AU2133 to Ap^r
AU2145	*frt mCherry-DnaN,* P_{lac}*-tetR-mCerulean frt* at *galK, tetO240::gen* at *ter3, ΔaraBAD frt (araC+), pZ63*	pZ63 × AU2133 to Ap^r
AU2155	*frt mCherry-DnaN,* P_{lac}*-tetR-mCerulean frt* at *galK, tetO240::gen* at *ter3, ΔaraBAD frt (araC+), ΔmatP::cat, pBAD24*	AU2143 × P1.SN205 to Cm^r Ap^r
AU2157	*frt mCherry-DnaN,* P_{lac}*-tetR-mCerulean frt* at *galK, tetO240::gen* at *ter3, ΔaraBAD frt (araC+), ΔmatP::cat, pZ63*	AU2145 × P1.SN205 to Cm^r Ap^r
ENOX5.114	*mukB-mCherry frt, parC-mYPet frt,* P_{lac}*-tetR-mCerulean-kan* at *galK*	This work
ENOX5.130	*tetO240::gen* at *ori1,* P_{lac}*-tetR-mCerulean frt* at *galK, mukB-mCherry frt, parC-mYPet-kan*	4
ENOX5.178	*parC-mYPet frt, tetO240::gen* at *ori1,* P_{lac}*-tetR-mCerulean frt* at *galK, mukB(E1407Q)mCherry-kan*	This work
ENOX5.212	*mCherry-DnaN,* P_{lac}*-tetR-mCerulean frt* at *galK, tetO240::gen* at *ter3*	This work
KK56	*tetO240::gen* at *ori1,* P_{lac}*-tetR-mCerulean frt* at *galK, mukB-mCherry frt, parC-mYPet-kan, ΔmatP::cat*	ENOX5.130 × P1.SN205 to Cm^r
KK57	*mukB-mCherry frt, parC-mYPet frt,* P_{lac}*-tetR-mCerulean-kan* at *galK, tetO240::gen* at *ter3*	ENOX5.114 × P1.RRL189 to Gm^r
KK58	*mukB-mCherry frt, parC-mYPet frt,* P_{lac}*-tetR-mCerulean-kan* at *galK, tetO240::gen* at *ter3, ΔmatP::cat*	KK57 × P1.SN205 to Cm^r
RRL80	*mukB(D1406A)-gfp-cat*	2
RRL149	*ΔmukB::kan, pKD46*	This work
RRL189	*lacO240::hyg* at *ori1, tetO240::gen* at *ter3,* P_{lac}*-lacI-mCherry frt* at *leuB,* P_{lac}*-tetR-mCerulean frt* at *galK*	5
SN53	*matP-3XFlag-kan*	This work
SN54	*mukB-3XFlag-kan*	This work
SN154	*mukB(E1407Q)-3XFlag-kan, pKD46*	This work

SN156	*mukB-3XFlag-kan, ΔmatP::cat*	SN54 × P1.SN205 to Cm^r
SN182	*lacO240::hyg* at *ori1, tetO240::gen* at *ter3, P_{lac}-lacI-mCherry frt* at *leuB, P_{lac}-tetR-mCerulean frt* at *galK, mukB-mYPet-kan*	RRL189 × P1.Ab16 to Km^r
SN192	*lacO240::hyg* at *ori1, tetO240::gen* at *ter3, P_{lac}-lacI-mCherry frt* at *leuB, P_{lac}-tetR-mCerulean frt* at *galK, mukB-mYPet frt*	Derivative of SN182, *kan* removed via pCP20
SN201	*mukB(D1406A)-3XFlag-kan, pKD46*	*gfp-cat* in RRL80 replaced by *3XFlag-kan* via λred recombination
SN205	*ΔmatP::cat*	AB1157 × P1.*ΔmatP::cat* to Cm^r. 6
SN209	*mukB(E1407Q)-3XFlag-kan, pRC7(mukB+)*	*pRC7(mukB+)* × SN154 to Ap^r
SN211	*mukB(D1406A)-3XFlag-kan, pRC7(mukB+)*	*pRC7(mukB+)* × SN201 to Ap^r
SN245	*mukB(E1407Q)-3XFlag-kan*	AB1157 × P1.SN209 to Km^r
SN247	*mukB(D1406A)-3XFlag-kan*	AB1157 × P1.SN211 to Km^r
SN249	*ΔmatP::cat, mukB(E1407Q)-3XFlag-kan*	SN205 × P1.SN209 to Km^r
SN299	*lacO240::hyg* at *ori1, tetO240::gen* at *ter3, P_{lac}-lacI-mCherry frt* at *leuB, P_{lac}-tetR-mCerulean frt* at *galK, ΔmatP::cat*	RRL189 × P1.SN205 to Cm^r
SN302	*lacO240::hyg* at *ori1, tetO240::gen* at *ter3, P_{lac}-lacI-mCherry frt* at *leuB, P_{lac}-tetR-mCerulean frt* at *galK, mukB-mYPet frt, ΔmatP::cat*	SN192 × P1.SN205 to Cm^r
SN311	*lacO240::hyg* at *ori1, tetO240::gen* at *ter3, P_{lac}-lacI-mCherry frt* at *leuB, P_{lac}-tetR-mCerulean frt* at *galK,* mukB(E1407Q)-mYPet-kan	RRL189 × P1.Ab198 to Km^r
SN317	*lacO240::hyg* at *ori1, tetO240::gen* at *ter3, P_{lac}-lacI-mCherry frt* at *leuB, P_{lac}-tetR-mCerulean frt* at *galK, ΔmatP::cat,* mukB(E1407Q)-mYPet-kan	SN299 × P1.Ab198 to Km^r
SN399	*lacO240::hyg* at *ori1, tetO240::gen* at *ter3, P_{lac}-lacI-mCherry frt* at *leuB, P_{lac}-tetR-mCerulean frt* at *galK, mukB-mYPet-kan, matPΔC20-cat*	SN182 × P1.AU2129 to Cm^r
**Other genetic backgrounds**		
BTH101	*F‘, cya-99, araD139, galE15, galK16, rpsL1 (*Str^r*), hsdR2, mcrA1, mcrB1, relA1*	Gift from D. Jakimowicz
C3013I	MiniF *lysY $lacI^q$*(Cm^r) */ fhuA2 lacZ::T7 gene1 [lon] ompT gal sulA11 R(mcr-73::miniTn10--*Tc^s*)2 [dcm] R(zgb-210::Tn10--*Tc^s*) endA1 Δ(mcrC-mrr) 114::IS10*	NEB
DHM1	*F', cya-854, recA1, endA1, gyrA96 (*Nal^r*), thi1, hsdR17, spoT1, rfbD1, glnV44(AS)*	7
FK01	C3013I derivative *mukB-3XFlag-kan*	C3013I × P1.SN54 to Km^r
MG1655	*F− rph-1 ara+*	1
RRL386	MG1655 derivative *ΔaraBAD::cat (araC+)*	This work

SN361	BTH101 derivative Δ*matP::cat*	BTH101 × P1.SN205 to Cm^r

[a]The abbreviations *kan*, *cat*, *gen*, and *hyg* refer to insertions conferring resistance to kanamycin (Km^r), chloramphenicol (Cm^r), gentamycin (Gm^r) and hygromycin B (Hyg^r). Ap^r, Str^r and Nal^r refer to ampicillin, streptomycin and nalidixic acid resistance, respectively. Tc^s refers to tetracyclin sensitivity. *frt* refers to the FLP site-specific recombination site.

Supplementary Table 2. Plasmids

Plasmid	Vector	Insert	Primers	Primer	Reference
pUT18C					8
pKNT25					7
pSN19	pKNT25	Hinge	THHingeForw (XbaI) cctctagagatgtctctttccgatagcgt	THHingeRev (KpnI) ccggtaccggctcacgctgctgctggtta	This study
pSN20	pKNT25	MatP	THMatPForw (XbaI) cctctagagatgaaatatcaacaacttgaaaa	THMatPRev2 (KpnI) ccggtaccggttccttacccagcaatgcct	This study
pSN24	pUT18C	Hinge	THHingeForw (XbaI)	THHingeRev (KpnI)	This study
pSN25	pUT18C	MatP	THMatPForw (XbaI)	THMatPRev2 (KpnI)	
pSN43	pKNT25	MatPΔ20C	THMatPForw (XbaI)	THMatPΔ20CRev (KpnI) ccggtaccgtctctttgttttccgcatctt	This study
pSN44	pKNT25	MatPΔ18C	THMatPForw (XbaI)	THMatPΔ18CRev (KpnI) ccggtaccgggtatttctctttgttttccgca	This study
pSN55	pUT18C	MatPΔ20C	THMatPForw (XbaI)	THMatPΔ20CRev (KpnI)	This study
pSN57	pUT18C	MatPΔ18C	THMatPForw (XbaI)	THMatPΔ18CRev (KpnI)	This study
pFK01	pKZ02	MukB-Flag	ForBamHI-FLAG-stop-SacI gatcccgactataaagatgacgatgacaagtaagagct	RevBamHI-FLAG-stop-SacI cttacttgtcatcgtcatctttatagtcgg	This study
pKZ02	pET21a	MukB	MukB For NheI ttatgctagcattgaacgcggtaaatttcgc	MukB rev(BamHI) ttatggatccgcactcgcctgagaaggcgcttc	This study
pKZ03	pET21a	MukE	MukE For NheI ttatgctagcccggttaagctggcgcagg	MukE rev(BamHI) ttatggatccgcttcttcctctccgctatctgg	This study
pKZ05	pET21a	$MukB^{EQ}$	MukB For NheI	MukB rev(BamHI)	This study
pKZ06	pET28a	MukF	MukF For NheI ttatgctagcagtgaattttcccagacagtccc	MukF rev(BamHI) ttatggatccttatcaatatttgtcgatgacatgcgcctg	This study
pKZ11	pET21a	MatPΔ18C	MatP NheI For ttatcagctagcaaatatcaacaacttgaa	MatP delC BamHI Rev ttatcaggatccaagtatttctctttgttttc	This study
pKZ12	pET21a	Hinge	Hinge NdeI For ctatgcatatgtctctttccgatagcgtgt	Hinge BamHI Rev ctatgggatcctcacgctgctgctggttatc	This study
pKZ13	pET21a	Hinge-Flag	Hinge NdeI For	Hinge BamHI FLAG Stop ctatgggatcctcttatgccttgtcatcgtcatctttatagtctgctgcacgctgctgctggttatc	This study
pZ63	pBAD24	ParC CTD			Zawadzki et al., unpublished data

Supplementary Methods

Bacterial strains, plasmids and growth

Bacterial strains and plasmids are listed in Supplementary Tables 1 and 2. Fusion of genes with fluorescent tags used λRed recombination[9]. Gene loci were transferred by phage P1 transduction[10] to generate the final strains. Where multiple insertions of modified genes were required the *kan* and *cat* genes were removed using site-specific recombination induced by expression of the Flp recombinase from plasmid pCP20[9]. Chromosomal loci were visualised by the fluorescence repressor-operator system[11–13]. The replication origin region was tagged by insertion of *lacO* or *tetO* arrays (240 copies) 15 kb counter-clockwise of *oriC* (*ori1*). The replication terminus region had *tetO* arrays inserted 50 kb clockwise of *dif* (*ter3*). LacI-mCherry and TetR-mCerulean were expressed from LacI-controlled genes in the chromosome[5] and enabled visualisation of the arrays. In cells with both *ori1* and *ter3* arrays appropriate levels of fluorescent repressors were expressed without the addition of IPTG, because LacI is titrated out by the *lacO* arrays, leading to modest derepressed expression from the LacI-controlled fluorescent LacI and TetR repressor genes. In strains with just the *tetO* arrays at *ori1* or *ter3*, IPTG was used to induce fluorescent repressor expression. pCTD is a derivative of pBAD24 from which the C-terminal domain of ParC is expressed upon addition of arabinose (pZ63 plasmid, Supplementary Table 2). pRC7(MukB$^+$) is an unstable low copy plasmid that encodes an IPTG-inducible wild type *mukB* gene and was present in *mukB* mutants to aid preparation of phage P1 lysates. Strains had a generation time of ~170 min at 30°C in M9 gycerol, and cell length distributions (in μm ± s. d.) as follows: 2.95 ± 0.13 (SN182), 3.13 ±

0.19 (SN302), 3.24 ± 0.10 (SN311), 3.25 ± 0.05 (SN317), 3.30 ± 0.19 (SN335), 3.14 ± 0.04 (SN400), 3.48 ± 0.07 (ENOX5.130), 3.46 ± 0.11 (KK56), 3.58 ± 0.06 (KK57), 3.61 ± 0.16 (KK58), 4.30 ± 0.34 (ENOX5.178). Flow cytometry analysis of steady state, 'run-out' and stationary phase cultures (Figure S9) showed that wild type and *ΔmatP* cells had very similar cell cycle profiles, with ~20% of steady state cells not having initiated replication, and with initiation and termination of DNA replication occurring in the same cell, all consistent with previously reported B, C and D periods[12]. Δ*mukB* cells had fewer cells with a single chromosome and a few more 4-chromosome cells in the run-outs. This is likely a consequence of a population of cells that had failed to segregate their chromosomes, generating an anucleate cell population and cells containing 2 unsegregated chromosomes, which after replication give the 4-chromosome population. As a consequence of this, the fraction of Δ*mukB* cells containing two *ter3* sites in snapshots (Fig. S1) does not reflect the increased cohesion observed in time-lapse analysis. Cells expressing fluorescent ParC derivatives and CTD had similar flow cytometry profiles to the wild type strain. These data, and the interpretation that Δ*matP*, Muk^- and pCTD over-expressing cells used here have close to wild type cell cycle parameters during 30° C growth in minimal glycerol medium, are corroborated by the analysis of numbers and positions of *ori1* and *ter3* in the strains used (Figure S1).

ChIP-seq data analysis

All reads were adapter removed and trimmed to 40 bp using trimmomatic[14]. Reads were mapped to the NC_000913.2 genome (MG1655), by bowtie v.

2.2.2[15] with parameters -N 0 --sensitive --minins 130 --maxins 780 -q --no-mixed --no-discordant --no-unal. All samples were sequenced in triplicates and at least 3 Million reads per replicate were uniquely mapped to the genome. Peaks were identified by MACS v2.0.10.20131216[16] using a maximum of 20 reads per unique position. Only highly enriched (greater than 2-fold enrichment over background) and highly significant (–log10 q value of greater than 30) peaks were used for the analysis, yielding 13 (MukB^{DA}), 3 (Δ*matP* MukB^{EQ}), 43 (MukB^{EQ}), 147 (MukB) and 375 (MatP) significant peaks, which are represented on the Circos plot[17] Supplementary Fig. 4. For direct comparison of enriched regions across samples in Fig. 3b and Supplementary Fig. 4a, samples were normalized to background reads as described before[18]. Background reads were summed for each sample excluding any significantly enriched regions as detected by MACS. Furthermore, due to the strong decrease of reads from *oriC* to *dif* site and the vast differences in enrichment, samples were weighted at each position for the global trend in background enrichment. The weights for detrending were calculated by fitting a locally-weighted polynomial regression (LOWESS) to the read density of each sample using the lowess function in R, with a span of 0.2 and the inverse of the fitted values were scaled to the maximum value. For visual representation, lines were smoothed using the natural cubic spline fit with 10 degrees of freedom. *De novo* Motif search was conducted by MEME-ChIP[19] using the peak center of the 43 significantly enriched MukB^{EQ} peaks extended by 100bp in both directions. For the Fig. 3a, aligned reads were normalized to a total of 1 million reads using Create a BedGraph of genome coverage, version 0.1.0[20], with the Galaxy platform[21–23]. The obtained bedgraph files were

visualized and processed using IGB browser[24]. The median bedgraph files of the triplicates were generated for Flag-tagged (IP) and untagged strains (Mock), then the bedgraph files of the ratio IP/Mock were created. Mock samples used are as follow: AB1157 grown at 37°C for MatP-Flag and MukB-Flag; AB1157 grown at 22°C for $MukB^{EQ}$ and $MukB^{DA}$; AB1157 Δ*matP* (SN205) grown at 22°C for $MukB^{EQ}$ Δ*matP*.

Protein purification

MukF, MukB, $MukB^{EQ}$, Hinge, ParC, MatPΔC18 and MukE co-expressed or not with MukF, were all 6xhis-tagged and expressed from pET vectors (Supplementary Table 2) in strain C3013I (NEB) or FK01 in the case of $MukB^{EQ}$. 2 L cultures were grown in LB with carbenicilin (100 μg ml^{-1}) at 37°C to A_{600}~0.6 and induced by adding IPTG to a final concentration 0.4 mM. After 2 hours at 30°C, cells were harvested by centrifugation at 6,747g for 20 min, resuspended in 30ml lysis buffer (50 mM HEPES pH 7.5, 300 mM NaCl, 5%glycerol, 10 mM imidazole) supplemented with 1 tablet of protease inhibitor (PI), and homogenized. Cell debris was removed by centrifugation at 20,400g for 20 min and clear cell lysates were mixed with 5 ml equilibrated TALON Superflow resin, poured into a column, then washed with 10 X volume of washing buffer (50 mM HEPES pH 7.5, 300 mM NaCl, 5% glycerol, 25 mM imidazole, PI). Bound proteins were eluted in elution buffer (50 mM HEPES pH 7.5, 300 mM NaCl, 5% glycerol, 250 mM imidazole).

For MukB and Hinge purifications, the fractions from TALON were diluted to 100 mM NaCl and injected to HiTrapTM Heparin HP column (GE Healthcare) pre-equilibrated with Buffer A (50 mM HEPES pH 7.5, 100 mM

NaCl, 10% glycerol, 1 mM EDTA, 1 mM DTT), then the column was washed at 1 ml/min flow rate until constant UV280. Purified fractions were eluted with a gradient 100 – 1,000 mM NaCl of Buffer A. For $MukB^{EQ}$, fractions from TALON were mixed with 100μl of Anti-FLAG M2 Affinity gel (Sigma Aldrich) and incubated for 1h at 4°C prior to dilution and injection to HiTrapTM Heparin HP column. For MukE and MukF purifications, fractions from Talon were diluted and injected to HiTrap DEAE FF column (GE healthcare) pre-equilibrated in buffer A. Purified fractions were eluted with a gradient 100 - 1,000 mM NaCl of buffer A. For ParC and MatPΔC18 purifications, the pooled Talon fractions were loaded directly on the HiTrapTM Heparin HP column pre-equilibrated with Buffer B (50 mM HEPES pH 7.5, 300 mM NaCl, 10% glycerol, 1 mM EDTA, 1 mM DTT) and eluted with gradient from 300 to 1,000 mM NaCl of Buffer B. Purity and concentration of pooled fractions were determined using SDS PAGE and a Nanodrop spectophotometer. Proteins were aliquoted and stored at -20°C in 10% glycerol.

ATP hydrolysis assays

ATP hydrolysis was analysed using an ENZCheck Phosphate Assay Kit (Life Technologies) in 150 μl samples containing standard reaction buffer supplemented with 2 mM of ATP and 30-50 mM NaCl in a BMG Labtech PherAstar FS plate reader at 25°C. The protein concentrations were: MukB/$MukB^{EQ}$ 0.5 μM, MukE 2.5 μM and MukF 1.25 μM. The results were computed using MARS data analysis software. Quantitation of phosphate release was determined using the extinction coefficient of 11,200 $M^{-1}cm^{-1}$ for the phosphate-dependent reaction at 360 nm at pH 7.0.

Flow cytometry

Flow cytometry was performed as described in ref. 25, except cells were grown in M9 glycerol at 30°C and for run-out experiments the cells were incubated in cephalexin and rifampicin for 4 hours. Data were analyzed using FlowJo software.

Western blot analysis of ParC-CTD expression

Strains were grown to an A_{600} of 0.1 at 30°C in M9 glycerol. Arabinose was added to a final concentration of 0.2% and cells were incubated for a further 60 min at 30°C. The equivalent number of cells as 2 ml at A_{600} 0.1 was harvested and resuspended in 60 µl of protein loading buffer (NEB) and boiled for 10 min. 7 µl samples were loaded onto an SDS-PAGE gel. Western blotting was performed using the iBlot dry blotting system (Invitrogen). Membranes were blocked with TBS, Tween 0.2%, milk 1% for 1 hour at room temperature and incubated with anti-ParC[26] diluted 1:10,000 in TBS, Tween 0.2%, milk 1% at 4°C overnight. Membranes were washed for 3 × 10 min in TBS Tween 0.2% at room temperature and then incubated with anti-rabbit-HRP diluted 1:10,000 in TBS Tween 0.2%. Membranes were washed again 3 × 10 min in TBS Tween 0.2% at room temperature. Peroxidase activity on the membrane was revealed using Supersignal WestPico chemiluminescent kit (ThermoScientific) and imaged using a ChemiDoc system (Bio-rad).

Supplementary References

1. Bachmann, B. J. Derivations and genotypes of some mutant derivatives of Escherichia coli K-12. *Escherichia Coli Salmonella Cell. Mol. Biol. 2nd Ed ASM Press Wash. DC* 2460–2488 (1996).
2. Badrinarayanan, A., Reyes-Lamothe, R., Uphoff, S., Leake, M. C. & Sherratt, D. J. In Vivo Architecture and Action of Bacterial Structural Maintenance of Chromosome Proteins. *Science* **338,** 528–531 (2012).
3. Badrinarayanan, A., Lesterlin, C., Reyes-Lamothe, R. & Sherratt, D. The Escherichia coli SMC Complex, MukBEF, Shapes Nucleoid Organization Independently of DNA Replication. *J. Bacteriol.* **194,** 4669–4676 (2012).
4. Nicolas, E. *et al.* The SMC Complex MukBEF Recruits Topoisomerase IV to the Origin of Replication Region in Live Escherichia coli. *mBio* **5,** e01001–13 (2014).
5. Reyes-Lamothe, R., Possoz, C., Danilova, O. & Sherratt, D. J. Independent Positioning and Action of Escherichia coli Replisomes in Live Cells. *Cell* **133,** 90–102 (2008).
6. Mercier, R. *et al.* The MatP/matS site-specific system organizes the terminus region of the E. coli chromosome into a macrodomain. *Cell* **135,** 475–485 (2008).
7. Karimova, G., Dautin, N. & Ladant, D. Interaction Network among Escherichia coli Membrane Proteins Involved in Cell Division as Revealed by Bacterial Two-Hybrid Analysis. *J. Bacteriol.* **187,** 2233–2243 (2005).
8. Karimova, G., Ullmann, A. & Ladant, D. Protein-protein interaction between Bacillus stearothermophilus tyrosyl-tRNA synthetase subdomains

revealed by a bacterial two-hybrid system. *J. Mol. Microbiol. Biotechnol.* **3,** 73–82 (2001).

9. Datsenko, K. A. & Wanner, B. L. One-step inactivation of chromosomal genes in Escherichia coli K-12 using PCR products. *Proc. Natl. Acad. Sci. U. S. A.* **97,** 6640–6645 (2000).

10. Thomason, L. C., Costantino, N. & Court, D. L. in *Current Protocols in Molecular Biology* (2007). at <http://onlinelibrary.wiley.com/doi/10.1002/0471142727.mb0117s79/abstract>

11. Lau, I. F. *et al.* Spatial and temporal organization of replicating Escherichia coli chromosomes. *Mol. Microbiol.* **49,** 731–743 (2003).

12. Wang, X., Possoz, C. & Sherratt, D. J. Dancing around the divisome: asymmetric chromosome segregation in Escherichia coli. *Genes Dev.* **19,** 2367–2377 (2005).

13. Wang, X., Liu, X., Possoz, C. & Sherratt, D. J. The two Escherichia coli chromosome arms locate to separate cell halves. *Genes Dev.* **20,** 1727–1731 (2006).

14. Bolger, A. M., Lohse, M. & Usadel, B. Trimmomatic: a flexible trimmer for Illumina sequence data. *Bioinformatics* **30,** 2114–2120 (2014).

15. Langmead, B., Trapnell, C., Pop, M. & Salzberg, S. L. Ultrafast and memory-efficient alignment of short DNA sequences to the human genome. *Genome Biol.* **10,** R25 (2009).

16. Zhang, Y. *et al.* Model-based analysis of ChIP-Seq (MACS). *Genome Biol.* **9,** R137 (2008).

17. Krzywinski, M. *et al.* Circos: an information aesthetic for comparative genomics. *Genome Res.* **19,** 1639–1645 (2009).
18. Diaz, A., Park, K., Lim, D. A. & Song, J. S. Normalization, bias correction, and peak calling for ChIP-seq. *Stat. Appl. Genet. Mol. Biol.* **11,** Article 9 (2012).
19. Machanick, P. & Bailey, T. L. MEME-ChIP: motif analysis of large DNA datasets. *Bioinforma. Oxf. Engl.* **27,** 1696–1697 (2011).
20. Quinlan, A. R. & Hall, I. M. BEDTools: a flexible suite of utilities for comparing genomic features. *Bioinformatics* **26,** 841–842 (2010).
21. Blankenberg, D. *et al.* in *Current Protocols in Molecular Biology* (2010). at <http://onlinelibrary.wiley.com/doi/10.1002/0471142727.mb1910s89/abstract>
22. Giardine, B. *et al.* Galaxy: a platform for interactive large-scale genome analysis. *Genome Res.* **15,** 1451–1455 (2005).
23. Goecks, J., Nekrutenko, A., Taylor, J. & Galaxy Team. Galaxy: a comprehensive approach for supporting accessible, reproducible, and transparent computational research in the life sciences. *Genome Biol.* **11,** R86 (2010).
24. Nicol, J. W., Helt, G. A., Blanchard, S. G., Raja, A. & Loraine, A. E. The Integrated Genome Browser: free software for distribution and exploration of genome-scale datasets. *Bioinforma. Oxf. Engl.* **25,** 2730–2731 (2009).
25. Lesterlin, C., Ball, G., Schermelleh, L. & Sherratt, D. J. RecA bundles mediate homology pairing between distant sisters during DNA break repair. *Nature* **506,** 249–253 (2014).

26. Hayama, R. & Marians, K. J. Physical and functional interaction between the condensin MukB and the decatenase topoisomerase IV in Escherichia coli. *Proc Natl Acad Sci U S A* **107,** 18826–18831 (2010).
